# NMR structural analysis of the yeast cytochrome *c* oxidase subunit Cox13 and its interaction with ATP

**DOI:** 10.1186/s12915-021-01036-x

**Published:** 2021-05-10

**Authors:** Shu Zhou, Pontus Pettersson, Markus L. Björck, Hannah Dawitz, Peter Brzezinski, Lena Mäler, Pia Ädelroth

**Affiliations:** 1 Department of Biochemistry and Biophysics, Stockholm University, Stockholm, Sweden; 2 Current address: High Magnetic Field Laboratory, Hefei Institutes of Physical Science, Chinese Academy of Sciences, Hefei, China

**Keywords:** ATP, Membrane protein, NMR, Solution structure

## Abstract

**Background:**

Mitochondrial respiration is organized in a series of enzyme complexes in turn forming dynamic supercomplexes. In *Saccharomyces cerevisiae* (baker’s yeast), Cox13 (CoxVIa in mammals) is a conserved peripheral subunit of Complex IV (cytochrome *c* oxidase, Cyt*c*O), localized at the interface of dimeric bovine Cyt*c*O, which has been implicated in the regulation of the complex.

**Results:**

Here, we report the solution NMR structure of Cox13, which forms a dimer in detergent micelles. Each Cox13 monomer has three short helices (SH), corresponding to disordered regions in X-ray or cryo-EM structures of homologous proteins. Dimer formation is mainly induced by hydrophobic interactions between the transmembrane (TM) helix of each monomer. Furthermore, an analysis of chemical shift changes upon addition of ATP revealed that ATP binds at a conserved region of the C terminus with considerable conformational flexibility.

**Conclusions:**

Together with functional analysis of purified Cyt*c*O, we suggest that this ATP interaction is inhibitory of catalytic activity. Our results shed light on the structural flexibility of an important subunit of yeast Cyt*c*O and provide structure-based insight into how ATP could regulate mitochondrial respiration.

**Supplementary Information:**

The online version contains supplementary material available at 10.1186/s12915-021-01036-x.

## Background

The mitochondrial electron transport chain (ETC) is comprised of a series of enzyme complexes (I–IV) where electrons are transferred through a series of electron donors and acceptors, coupled to the generation of a proton gradient across the mitochondrial inner membrane. This gradient is used for energy-requiring processes, such as ATP synthesis by Complex V (ATP synthase). Complex IV (cytochrome *c* oxidase, Cyt*c*O) is the terminal enzyme of the ETC and catalyzes the reduction of oxygen to water coupled to the translocation of protons. Cyt*c*O is comprised of three mitochondrially encoded core subunits and a number of nuclear-encoded small and peripheral subunits (for recent reviews, see [1, 2]). The three core subunits, containing all the redox-active cofactors—Cu_A_, heme *a*, and the catalytic site consisting of heme *a*_3_ and Cu_B_—exhibit high conservation throughout many respiring organisms [3, 4]. The remaining subunits are present only in eukaryotes and their number varies; there are, e.g., ten (or eleven ([5, 6]) additional subunits in *Bos taurus* and eight (or nine, see [7]) in *Saccharomyces* (*S*.) *cerevisiae*. The function of the nuclear-encoded additional subunits within the complex is poorly understood, but most mitochondrial Cyt*c*Os possess homologs of most of them and some of them also have isoforms expressed differently depending on, e.g., tissue type [2]. For some of these supernumerary subunits, roles in assembly, stability, and regulation of the complex have been suggested. For instance, subunit IV in mammalian Cyt*c*Os, which corresponds to Cox5 in yeast, has isoforms differently expressed depending on oxygen tension [8] and has been implicated in ATP binding [1] and subunit Vb (Cox4 in yeast) has a binding site for Zn.

In the crystal structure of the dimeric bovine Complex IV, subunit VIa is located at the dimer interface [9], whereas in the cryo-EM structures of mammalian supercomplexes, it has a peripheral location [10, 11]. In the mammalian mitochondrial membrane, Complex IV is present (as a monomer) in different forms of supercomplexes as well as on its own in both monomeric and dimeric forms [12]. In *S. cerevisiae*, the corresponding subunit, Cox13, resolved to a varying degree in the recent cryo-EM structures of the III_2_IV_1-2_ supercomplex [7, 13] was confirmed to localize at a peripheral position without contact with the Complex III dimer. In the *S. cerevisiae* mitochondrial membrane, Complex IV is present mostly in a supercomplex with Complex III or as a monomer (see, e.g., [12, 14]), whereas the dimeric form was found (at a low level) in the purified Complex IV preparation [15].

In bovine mitochondria, subunit CoxVIa (the yeast Cox13 homolog) was suggested to house an allosteric ATP binding site at the N terminus residing in the mitochondrial matrix [16]. Bovine as well as human CoxVIa displays tissue-specific isoforms [16, 17] and might therefore be involved in fine-tuning the response of Complex IV to environmental factors.

*S. cerevisiae* Complex IV, where the nuclear-encoded subunits are amenable to genetic manipulation, can assemble and is active in the absence of Cox13 [18]. Cox13 together with Cox12 has been found to be easily lost from Complex IV and a Cox13-deficient form, found even in wildtype mitochondria [19], is associated with formation of reactive oxygen species [19]. It was shown that the activity of the Cox13-deficient Complex IV has an altered dependence on ATP [20]. Taanman et al. suggested that there are two allosteric ATP binding sites, one of which is present in Cox13, but in contrast to the bovine Cyt*c*O, the ATP binding site in yeast was suggested to locate to the C-terminal part of Cox13, residing in the intermembrane space (IMS).

In this study, we investigated the detailed role of *S. cerevisiae* Cox13. For this purpose, we expressed it separately from the Complex IV and determined its structure in detergent micelles using solution NMR. We then used NMR to localize a possible allosteric ATP binding site on Cox13 and found that residues at the C terminus of Cox13, which resides in the intermembrane space, interact with ATP. Based on our data, we modeled possible ways to achieve this allosteric site. We also studied the catalytic activity of Cyt*c*O from both wildtype and a Cox13-deletion strain of *S. cerevisiae*, which showed altered dependence on ATP. Possible mechanisms for the ATP effect on Cyt*c*O activity mediated by Cox13 are discussed. This study provides new structure-based insight into the function of Cox13 and the regulation of mitochondrial respiration.

## Results

### Characterization of the refolded Cox13 protein

**Fig. 1:**
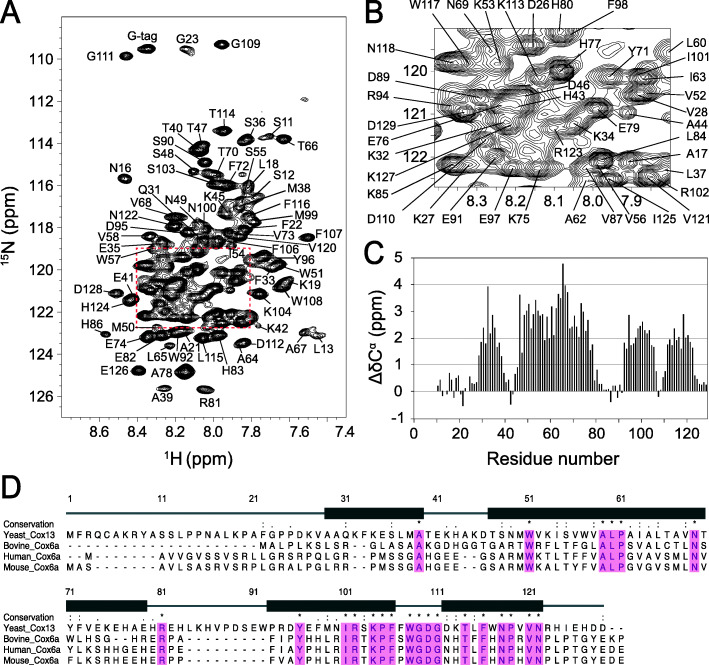
Backbone chemical shift assignment of Cox13 in DPC micelles. **a** 900 MHz 2D ^1^H-^15^N TROSY-HSQC spectrum of 0.4 mM [^15^N, ^13^C]-labeled Cox13 in 20 mM NaP_i_ pH 6.5, 50 mM L-Arg, 50 mM L-Glu, 1 mM DTT, and 30 mM DPC recorded at 40 °C. The assignment of each peak is given with residue one-letter code and sequence number. **b** For clarity, the central region of the spectrum is enlarged. **c** The Cα secondary chemical shifts of Cox13 in DPC micelles plotted versus the amino acid sequence. Consecutive positive secondary chemical shifts indicate α-helical secondary structure. **d** Protein sequence alignment for Cox13 in selected homologs. Positions of experimentally determined α-helices are indicated above the alignment. Pink shading indicates conserved residues across the homologs

In this study, Cox13, with a predicted mass of 15 kDa, was recombinantly overexpressed in *Escherichia coli.* Inclusion-body protein precipitate was refolded in the detergent dodecylphosphocholine (DPC). From a size-exclusion column (SEC), the Cox13-DPC complex eluted in a single peak (Additional file 1: Figure S1A), indicating that it was present in a single oligomeric state with an apparent mass of 55–60 kDa (see calibration inset in Additional file 1: Figure S1A). This size is consistent with a dimer of Cox13 (2×15 kDa) in a DPC micelle (~25 kDa [21]). The expected molecular mass and purity were confirmed by SDS-PAGE, where the Cox13 monomer runs at an apparent mass of 16 kDa and a fraction consistent with a dimer was also observed (Additional file 1: Figure S1B). Far-UV circular dichroism (CD) spectroscopy revealed that α-helix is the dominant structural element in refolded Cox13 protein (Additional file 1: Figure S1C). The Cox13-DPC complex was further analyzed by a two-dimensional (2D) heteronuclear single quantum correlation (HSQC) NMR experiment, and was found to have good spectral characteristics, and the expected dispersion of resonances typical for an alpha-helical protein [22, 23]. A transverse relaxation optimized spectroscopy (TROSY) type ^15^N-^1^H HSQC spectrum recorded at 900 MHz demonstrated that conducting solution NMR-based structural studies on Cox13 was feasible (Fig. 1).

### NMR resonance assignment

The backbone chemical shifts assignment of Cox13 was completed to 93% using a combination of six TROSY-type heteronuclear triple-resonance experiments with a [U-^15^N, ^13^C]-labeled sample (Additional file 1: Figure S2). The secondary structure of Cox13 in DPC micelles was determined using backbone secondary chemical shifts [24], which clearly demonstrate that Cox13 contains four α-helices (Fig. 1c): α1 (A29–A39), α2 (T48–E79), α3 (P93–F106), and α4 (D112–R123), which are separated by regions lacking well-defined secondary structure. Resonance assignments were extended using the combination of side-chain correlation experiments and nuclear Overhauser effect (NOE) experiments on differently labeled samples. Specifically, the assignment of approximately 79% of side-chain resonances was achieved (Additional file 1: Figure S3). The NOE cross-peak assignments were obtained by an iterative procedure using a combination of manual and automatic approaches. A total of 1324 intramolecular NOE distance restraints were extracted from the ^15^N and ^13^C-edited NOESY-HSQC spectra. Moreover, using a mixed sample with 50% [U-^15^N, ^13^C]-labeled protein and 50% unlabeled protein, ^15^N, ^13^C-filtered/edited NOESY experiments were carried out to detect NOE cross-peaks that are due to intermolecular interactions [25] (Additional file 1: Figure S4). The presence of intermolecular NOE cross-peaks in combination with the SEC profile indicated that Cox13 forms a dimer under our experimental conditions. Altogether, the collected data allowed us to determine a well-defined Cox13 dimer structure.

### Solution structure of Cox13 dimer in DPC micelles

**Fig. 2:**
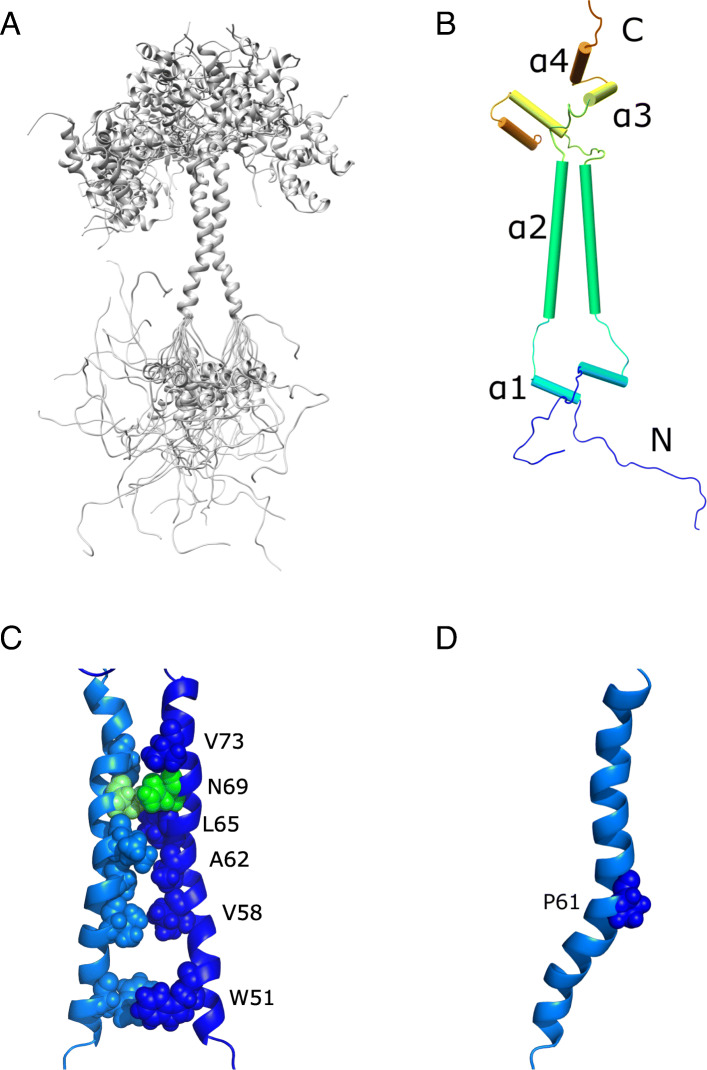
Solution structure of Cox13 in DPC micelles. **a** Backbone ribbon trace of the 15 lowest-energy structures determined by solution-state NMR. **b** Cylindrical representation of the Cox13 dimer structure. The four helices of a monomer are shown (see labels). **c** Clusters of stabilizing nonpolar interactions (shown are those between W51, V58, A62, L65, and V73 from each helix) at the dimer interface, and the polar interaction between the two N69, shown in green. **d** Residue P61 inducing the kink in the TM helix is shown in sphere representation

A final ensemble of the 15 lowest-energy structures calculated using the program CNS [26] representing the Cox13 dimer in DPC micelles is depicted in Fig. 2a. Summaries of the experimental constraints and structural statistics are given in the supplementary information (Additional file 1: Tables S1 and S2). Overall, the solution structure of the Cox13 dimer is characterized by the dimeric transmembrane (TM) entity, composed of the TM helix from each monomer, forming a two-helix bundle (Fig. 2b). Apart from the TM region (α2), each Cox13 monomer structure also contains three short helices, the α1-helix at the N terminus, and the α3- and α4-helices at the C terminus. The helices are connected by short- or medium-length loops. These helices do not have well-defined positions in the structure (Fig. 2), but a few distance restraints between the α3 and α4 helices were found, indicating that they interact, at least transiently. The high helicity of the Cox13 dimer structure is consistent with the CD spectrum and secondary chemical shift analysis (Additional file 1: Figure S1 and Fig. 1c).

The Cox13 dimer interface is well-defined from inter-molecular NOEs and its dimeric TM core is mainly formed by hydrophobic interactions, which include the residues W51, V58, A62, L65, T66, F72, and V73 (Fig. 2c and Additional file 1: Table S2). In addition, an interaction between N69 in each monomer appears to stabilize the dimer formation. Notably, residue P61 induces a kink in the TM helix (Fig. 2d), which is presumably involved in forming the bow-shaped structure observed for the Cox13 in the full III_2_IV_2_ supercomplex structure [7].

In summary, the solution structure of the Cox13 dimer in DPC micelles comprises a well-defined TM bundle induced by the interaction between the TM α2-helix from each monomer, while the positions of the other helices are less defined. NOEs were, however, found between the α3 and α4 helices indicating an interaction between them.

To better understand the position of Cox13 in micelles, we carried out paramagnetic relaxation enhancement (PRE) experiments by recording 2D TROSY-HSQC spectra in the presence of either the detergent-soluble 16-doxyl stearic acid (16-DSA) or water-soluble gadodiamide. From data obtained with the latter compound, the most affected (and thus solvent-exposed) regions of Cox13 is the random coil following the TM helix, as well as the C-terminal unstructured residues (Additional file 1: Figure S5). In comparison, the α3 and α4 helices have slightly higher intensity ratios than the flanking loops and are thus only partially solvent-exposed. 16-DSA data supports the gadodiamide results for the aforementioned regions by showing opposite trends in intensity ratios. As expected, detergent-embedded 16-DSA has a large effect on the residues of the transmembrane helix, but also interestingly on some residues in the N-terminal loop and on the α3 and α4 helices. The results thus indicate an interaction of α3 and α4 with detergent in the micelle-water interface. Together with gadodiamide’s overall modest attenuation of the signal intensity for the N-terminal loop and α1 helix, the results indicate that large parts of Cox13 fold back on and interact with the micelle. We conclude that the interaction with detergent contributes to the stability of the non-transmembrane helices α1, α3, and α4.

### Comparisons to Cox13 and homologs in full Cyt*c*O structures

**Fig. 3:**
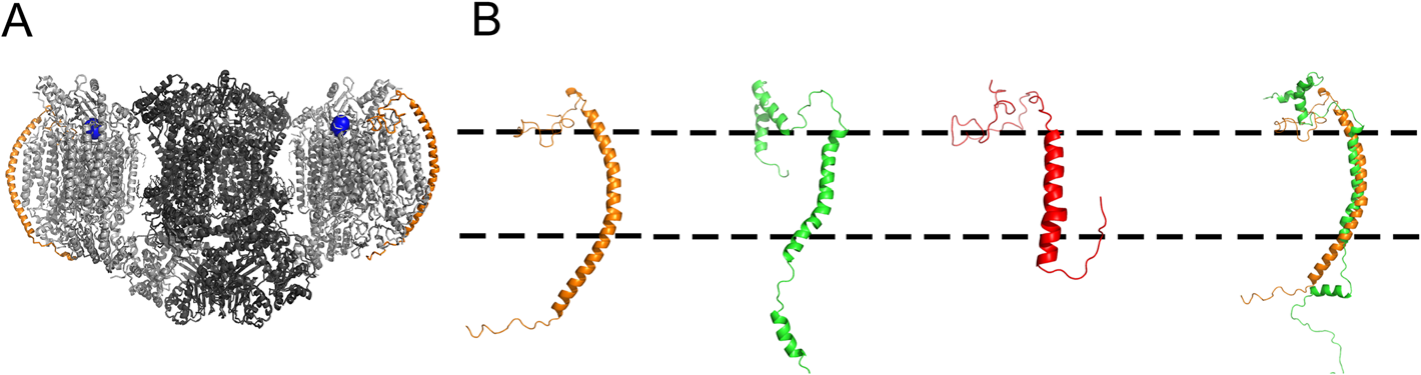
A comparison of the NMR structure of Cox13 from *S. cerevisiae*, the cryo-EM structure and the crystal structure of its *Bos taurus* homologous subunit VIa. **a**. The cryo-EM structure of the *S. cerevisiae* III_2_IV_2_ supercomplex (PDB ID: 6HU9 [7]). The Complex IV monomers are marked in light gray, the Complex III dimer in dark gray, the Complex IV electron entry site Cu_A_ as blue spheres (the size is exaggerated to emphasize the Cu), and Cox13 in orange. **b** A structural comparison of Cox13 from three different sources. The Cox13 from the III_2_IV_2_ *S. cerevisiae* complex in orange, one of the Cox13 monomers in the NMR (dimer) structure in green, and the *B. taurus* subunit VIa (from PDB ID: 1occ [9]) in red. On the right, the NMR structure of Cox13 is aligned with the same subunit from the cryo-EM structure. The approximate location of the membrane is marked by dashed lines

The Cox13 subunit was recently resolved in  a cryo-EM structure of the III_2_IV_2_ supercomplex from *S. cerevisiae* [7], which shows that it is localized at a peripheral position without contact with the Complex III dimer (Fig. 3a). A comparison of our NMR structure to the Cox13 structure in the III_2_IV_2_ complex (Fig. 3b) shows a similar overall topology, with a relatively well-aligned TM region, but significant differences in the overall structure. The largest differences are in the soluble domains, where the region occupying the intermembrane space (IMS) is mostly disordered in the supercomplex, whereas it folds into two short helices in our NMR structure. However, this is a very flexible region of the protein, which in the III_2_IV_2_ complex makes contacts with Cox3 and Cox12, discussed further below (and see [27]). Yeast Cox13 is in part homologous with bovine subunit VIa and is similarly positioned in relation to other Cyt*c*O subunits. In the crystal structure of the bovine Complex IV dimer, subunit VIa is located at the dimer interface [9], but there are no major differences to the subunit VIa structure in the monomeric Cyt*c*O form [28]. Comparing yeast Cox13 to the bovine subunit VIa (Fig. 3b), the largest differences are seen at the matrix-facing N-terminal region, where Cox13 has an organism-specific extension which is not present in subunit VIa.

In the yeast cryo-EM supercomplex structure, the Cox13 TM helix is significantly longer than in our NMR structure, and the TM region makes contact with Cox3, and the recently resolved (for specific conditions) respiratory supercomplex factor 2 (Rcf2) [27]. This applies to some of the residues that form the dimer interaction surface in our NMR structure (Fig. 2c), e.g., N69 and V73, which are in contact with Cox3, while V58 and W51 interact with Rcf2. It is interesting to note that the Rcf2 protein, which has been suggested to regulate supercomplex formation and Cyt*c*O activity [29–31], thereby could influence the propensity for Cox13 to induce dimerization of the Cyt*c*O, which then could lead to a “string” of supercomplexes, as suggested earlier [32].

#### Influence of nucleotides on the *Saccharomyces cerevisiae* Cyt*c*O catalytic activity

For both yeast and bovine Cyt*c*O, Cox13 (or equivalent) has been suggested to house an allosteric ATP binding site, but at different locations; yeast Cox13 was proposed to house such a site in the IMS [20] whereas in mammalian (bovine) mitochondria, subunit CoxVIa (equivalent to Cox13), was suggested to contain an allosteric nucleotide-binding site at the matrix-facing N terminus [16].

We purified wildtype *S. cerevisiae* Cyt*c*O with an engineered His-tag on Cox13 to ensure the presence of the subunit (as Complex IV in yeast shows a fraction lacking Cox13 [19]). We also purified the Cox13Δ-Cyt*c*O (with a Flag-tag on Cox6) in order to assess the influence of the Cox13 subunit on catalytic turnover. We observed no significant effect on the maximum catalytic activity of Cyt*c*O in the absence of Cox13, similar to previous results [20], but the relative activity (between wildtype and Cox13Δ-Cyt*c*O) varied between preparations and with buffer conditions (Additional file 1: Figure S6). ADP had small stimulatory effects for both variants. High phosphate concentrations had a small stimulatory effect in wildtype Cyt*c*O, which was increased in Cox13Δ-Cyt*c*O (Additional file 1: Figure S6). Addition of ATP also increased turnover activity in both wildtype and the Cox13Δ-Cyt*c*O (Additional file 1: Figure S6), and tentative fits indicate that ATP binds less tight (with tentative *K*_d_ in the mM range, qualitatively similar to a previous study [20]) and with a higher maximum activity in Cox13Δ-Cyt*c*O (Additional file 1: Figure S6BC). These (and previous [20]) data indicate a complex behavior with more than one ATP binding site on Cyt*c*O. In the previous study [20], the results were interpreted in terms of two regulatory ATP binding sites in Cyt*c*O, where one is stimulatory and one is inhibitory and located on Cox13.

**Fig. 4:**
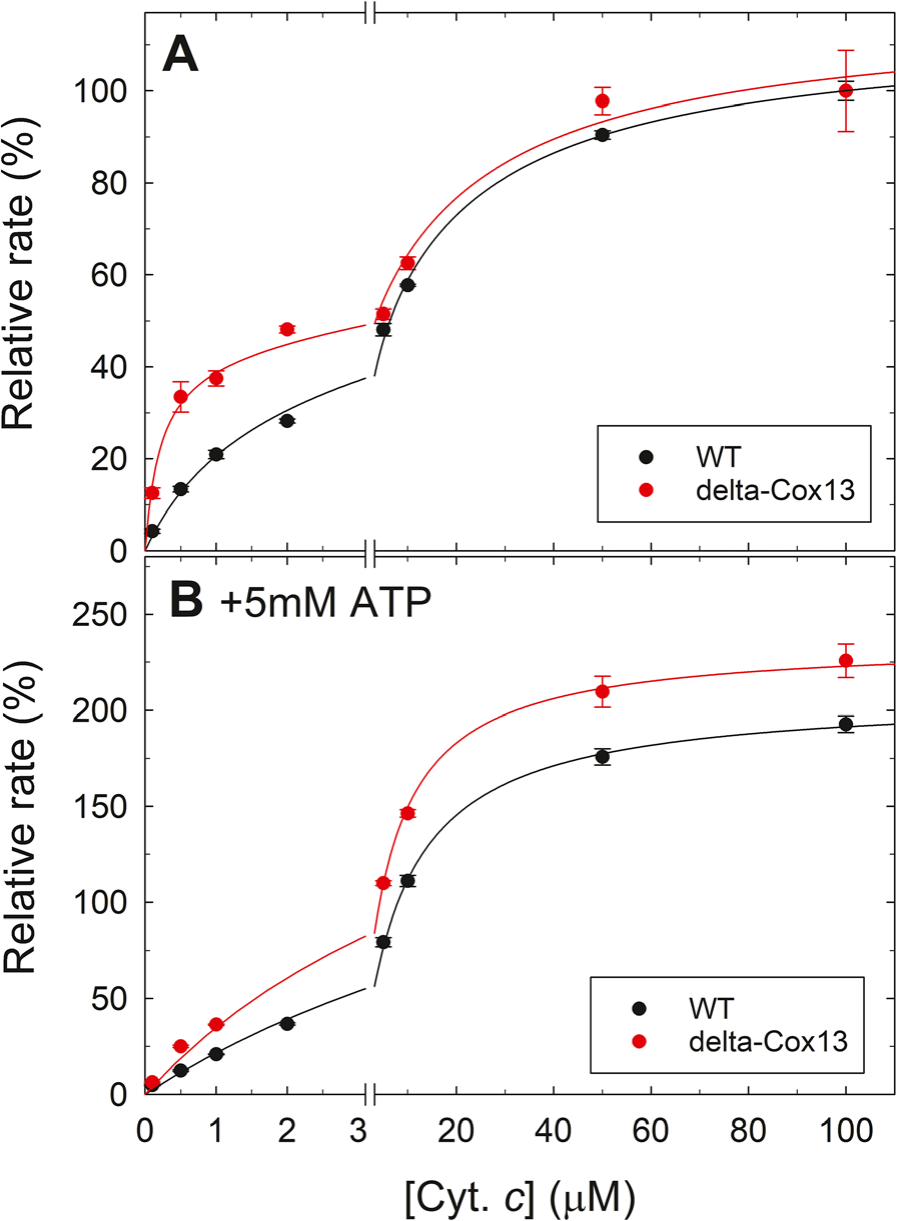
Turnover rate of wildtype and Cox13Δ-Cyt*c*O as a function of added yeast cyt. *c* in the absence (**a**) and presence (**b**) of 5 mM ATP. The turnover rate is expressed as relative to the maximum rate achieved (in e^-^/s) at 100 μM cyt. *c* in the absence of additions for both the wildtype and Cox13Δ-Cyt*c*O. For original data, see Additional file 1: Figure S7 and Additional file 2. Data points shown are averages and error bars are standard deviations (*n*=3). In **a**, the lines shown are fits to two hyperbolic functions with two *K*_m_s as follows: Wildtype (black line): V^1^: 40±10%, *K*_m_^1^: 1.3±0.6 μM, V^2^: 70±10%, *K*_m_^2^: 20±10 μM. Cox13Δ-Cyt*c*O (red line) V^1^: 43±7%, *K*_m_^1^: 0.2±0.1 μM V^2^: 74±7%, *K*_m_^2^: 20±10 μM. In **b,** the lines are fits to a simple Michaelis-Menten equation (one *K*_m_): for wildtype (black line) with V: 208±2%, *K*_m_: 8.6±0.3 μM and for Cox13Δ-Cyt*c*O (red line): 236±3%, *K*_m_: 5.8±0.3 μM. Experimental conditions: 25 mM KP_i_ pH 7.0, 0.035% DDM, 10 mM ascorbate, and 100 μM TMPD

The data presented above were not easily interpreted, and in the bovine Cyt*c*O, it has been shown that the interaction between the electron donor cyt. *c* and Cyt*c*O is highly dependent on the presence of anions, including ATP and phosphate [33, 34], which could complicate titrations such as those described above. Therefore, we monitored the Cyt*c*O activity as a function of added yeast cyt. *c* in both the absence and presence of 5 mM ATP using a low-P_i_ buffer for both the wildtype and the Cox13Δ-Cyt*c*O (Fig. 4 and Additional file 1: Figure S7). In the wildtype Cyt*c*O, we fitted the data to two *K*_m_s for cyt. *c* with values of 1.3±0.6 μM and 20±10 μM (Fig. 4a), consistent with a previous study that also found two *K*_m_s ([35], but see also [36]). In the Cox13Δ-Cyt*c*O, the higher *K*_m_ interaction with cyt. *c* is very similar, i.e., 20±10 μM, while the lower *K*_m_ is lowered to 0.2±0.1 μM. In the presence of 5 mM ATP, there are two clear effects in the wildtype Cyt*c*O; first the low *K*_m_ cyt. *c* binding site is clearly inhibited (or lost) (similar to the effect observed in bovine Cyt*c*O [33]), and the data can be fitted with only one *K*_m_ of 8.6±0.3 μM (Fig. 4b and S7). Moreover, the overall maximum activity is stimulated to 208±2% (Fig. 4b). In Cox13Δ-Cyt*c*O in the presence of 5 mM ATP, the low *K*_m_ interaction with cyt. *c* is similarly lost with only one *K*_m_ at 5.8±0.3 μM (Fig. 4b). Also here, the maximum activity is increased, by a larger factor to 236±3%. Our data shows that the overall affinity for cyt. *c* is lowered in the presence of ATP in both wildtype and Cox13Δ-Cyt*c*O, but that the increase in the maximum rate is higher in Cox13Δ- than in wildtype Cyt*c*O. The stimulation of activity in the presence of phosphate is also higher in Cox13Δ-Cyt*c*O than in wildtype (see Additional file 1: Figure S6A). It is clear that there are several effects of ATP binding to Cyt*c*O and that removal of the Cox13 subunit impacts at least one of these effects; the maximum stimulation of activity. However, the removal of Cox13 has complex effects on the Cyt*c*O activity, discussed further below. There is a difference between our results for the effect of ATP on yeast Cyt*c*O/cyt. *c* interactions and those obtained for the bovine (or horse) Cyt*c*O/cyt. *c* interactions where ATP more clearly inhibits Cyt*c*O activity at low cyt. *c* [33, 34]. The reason for this difference is presently unknown, but could be related to the observation that in addition to binding Cyt*c*O, ATP binds and affects also horse cyt. *c* (see e.g. [37]), something that was suggested to not occur in yeast cyt. *c* [38].

#### Interaction of Cox13 with ATP and ADP

We addressed the question of whether or not ATP binds to Cox13 by NMR and CD titration experiments. We first studied ATP/Cox13 interaction by CD up to a molar ratio of ~20:1 (corresponding to 2.5 mM ATP and 135 μM protein), but measurements at higher ratios were prevented by ATP’s high absorbance. For the ATP/Cox13 ratios possible to assay in CD experiments, slight changes in secondary structure were observed (Additional file 1: Figure S9B). The changes indicate a small increase in helicity, as the absolute intensities of the peaks at 208 and 222 nm increased.

**Fig. 5:**
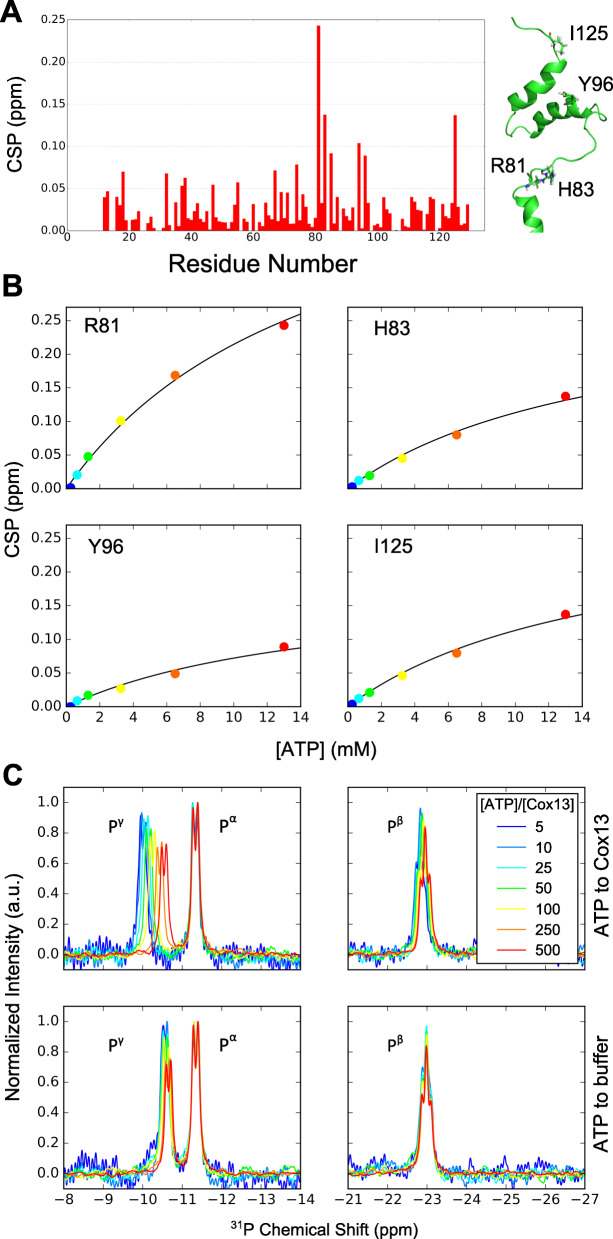
Interaction of Cox13 with ATP. **a** CSPs of Cox13 upon ATP addition, for ^15^N-HSQC spectra, see Additional file 1: Figure S8. CSPs as a function of primary sequence calculated from peak positions in ^15^N-HSQCs at the initial and final titration steps of [ATP]/[Cox13]=0 and 100, respectively (left). The residues with the largest CSPs are indicated as sticks on a representative structure from the NMR ensemble (right). **b** CSP as a function of ATP/Cox13-concentration ratio for the residues highlighted in **a**. The curves are in the form of Eq. 6 in [39] with globally fitted *K*_d_ = 15±2 mM. The fitted maximum CSPs for residues R81, H83, Y96, and I125 are 0.55±0.04, 0.29±0.02, 0.18±0.02, and 0.29±0.02 ppm, respectively. **c** Part of ^31^P spectra showing signals from the three phosphate groups of ATP in solutions with (upper panels) and without Cox13 (lower panels) at the concentration ratios given in the legend. The lower panels were obtained on a sample where ATP stock solution was added in the corresponding amounts to NMR buffer only. To ease shift change comparisons between signals from spectra with large ATP concentration differences, spectra have been normalized to the same P^α^ peak intensity

Next, the influence of ATP on ^15^N-labeled Cox13 was assayed by monitoring chemical shift changes in ^1^H-^15^N-HSQC spectra. Spectra were collected at six different concentrations of ATP (with the same protein concentration as in the CD experiments) resulting in ligand/protein ratios of 2:1, 5:1, 10:1, 25:1, 50:1, and 100:1 (Fig. 5a and Additional file 1: Figure S8), the highest ratio corresponding to [ATP]=13 mM. Higher ATP/Cox13 concentration ratios induced protein aggregation, possibly due to larger conformational changes in Cox13 induced by ATP. From the incremental addition of ATP to Cox13 in the NMR titrations, we observed gradual shift changes in resonance positions. A chemical shift perturbation (CSP) analysis across the sequence (Fig. 5a) revealed that the most affected residues are located in two distinct regions of the Cox13 structure; in the loop region immediately following the TM-helix (R81, H83, K85), and in the beginning of α3 (R94, Y96). A residue at the C terminus was also significantly affected by ATP addition (I125). Examples of CSPs as a function of the ligand/protein ratio are shown in Fig. 5b for four of the most affected residues. The higher CSPs suggest that these residues are in direct contact with ATP or are indirectly affected by the environmental changes produced by ATP binding, e.g., through conformational rearrangement of the protein. We note that although full saturation has not been achieved, the data indicates that at the highest concentration we see an onset of saturation for all residues. A global *K*_d_ of 15±2 mM was readily fitted to the data for all residues that displayed large CSPs, indicating that the changes in chemical shifts are consistent with one single binding event (Fig. 5).

To investigate the corresponding effects, those on ATP upon binding of the Cox13 protein, changes in ^31^P NMR spectra of ATP were monitored in a titration series of ATP to unlabeled Cox13. Significant ^31^P chemical shift changes were observed for the terminal, negatively charged phosphate group of ATP, with only minor changes for the central phosphate and no effect on the innermost one, indicating that electrostatic interactions are important for binding (Fig. 5c and Figure S9).

We also titrated ADP to Cox13 in the same way as for ATP, and the results are shown in Additional file 1: Figure S10. Overall, chemical shift changes suggest that ADP interacts with Cox13 in a similar manner as ATP, with the same residues being affected by the nucleotide. Also the fitted *K*_d_ values were very similar (17±2 mM for ADP vs 15±2 mM for ATP). Similarly as for ATP, the terminal phosphate in ADP was the most affected part of the molecule upon addition of Cox13. However, Cox13 was not observed to precipitate together with ADP at the final titration step, possibly indicating a difference in the interaction between Cox13 and the two nucleotides at higher ligand concentrations.

**Fig. 6:**
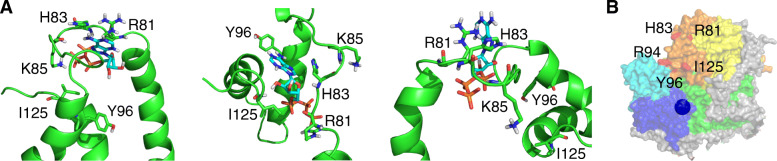
Cox13-ATP interaction models and location of the ATP binding site in the full yeast Cyt*c*O. **a** The three top-scoring Cox13-ATP interaction models from Haddock. Cartoon representation of the C terminus of Cox13, with ATP and residues discussed in the text displayed as sticks. **b** The location of identified ATP-binding residues (residues numbered and displayed in red) on the III_2_IV_2_ cryo-EM structure surface [7]. Only Complex IV (one monomer) is shown with Cox13 in orange, Cox2 and Cu_A_ (which sits below the surface) in blue, Cox1 in green, Cox3 in yellow, and Cox12 in cyan. The images were prepared in Pymol

In order to model how Cox13 interacts with ATP in our studies, the CSP data were used to direct docking of ATP to the Cox13 NMR structure. Due to the NMR ensemble’s large conformational heterogeneity, good binding scores were obtained in several Cox13 C-terminal structural arrangements (Fig. 6). The top-scoring models had extensive electrostatic contacts in common, between the negatively charged phosphate groups of ATP and the positively charged side-chains or polar backbone amide groups of residues in the loop preceding the α3 helix, e.g., R81, H83, and K85. The cluster with the best HADDOCK score (−91 ± 4) had well-defined electrostatic (−290 ± 40 kcal mol^-1^) and Van der Waals (−22 ± 4 kcal mol^-1^) energies. Three top-scoring models from this cluster are displayed in Fig. 6a. Although the top-scoring models never made simultaneous, direct contact with ATP using all residues with large CSPs, good overall docking scores could be obtained where different combinations of perturbed residues participated. This indicates either that the interaction can occur in several ways or that some residues with large CSPs are indirectly affected by ATP binding. Furthermore, it is possible that Cox13 forms other contacts with ATP in addition to those residues identified by NMR and ^1^H-^15^N-HSQC analysis. Our data is therefore most consistent with the presence of several possible Cox13-ATP complexes (for the isolated Cox13 subunit), but that the interactions are confined to the C-terminal region of Cox13, including a few charged residues.

## Discussion

It is clear from our data that the interaction between ATP and Cox13 occurs in the C-terminal region of Cox13, located in the intermembrane space. The bovine CoxVIa residues at the N terminus (matrix) suggested to be involved in ATP binding (Arg14 and Arg17) are not conserved in yeast Cox13, which could explain this difference [2]. However, the C terminus is highly conserved; specifically, the R81 and Y96 residues identified here to be in interaction with ATP are conserved in various species (Fig. 1d), suggesting that the binding of ATP to the Cox13 protein family may be a common feature. The conserved GDGXX [T/S] (where the first G is G109 and X is any amino acid) motif suggested by Taanman to be involved in the binding of ATP by Cox-13 [20] is in the same conserved region of the Cox13 protein (Fig. 1d) which also bears some similarity to the KXR/SXN ATP-binding motif in threonylcarbamoyl-AMP synthase [40, 41].

What could be the functional effect of binding ATP to Cox13 in the context of the entire Cyt*c*O complex? Based on the structural location (Fig. 6b) of Cox13 and our data showing effects on the low *K*_m_ for cyt. *c* in Cox13Δ-Cyt*c*O, we suggest that the Cox13 subunit is involved in forming the surface for electron donation, consistent with previous data suggesting that cyt. *c* docks in the vicinity of Cox13 [9, 42, 43] in the cleft lined by Cox1, Cox2, Cox3, and Cox12 (Fig. 6b). This binding site would then “open up” to yield a higher affinity for cyt. *c* in the absence of Cox13.

In the presence of 5 mM ATP, there are two effects on the interaction between cyt. *c* and Cyt*c*O (Figs. 4 and S7); the overall affinity is lowered, but the maximum activity is increased. Both these effects are seen also in the Cox13Δ-Cyt*c*O, but the overall rate increase is higher in the absence of Cox13. The effect of ATP on Cyt*c*O is thus consistent with a complex behavior with more than one interaction site with different effects, as previously suggested [20]. If we assume that there are two ATP binding sites on Cyt*c*O, one at an undefined location that leads to a lower affinity for cyt. *c* but a higher maximum activity, then our data is consistent with a second binding site for ATP with an inhibitory role on Cox13, since the overall rate stimulation by ATP is higher in its absence. Thus, ATP binding to Cox13 would be involved in regulating the mode (but not the affinity) of cyt. *c* binding to Cyt*c*O and thereby decrease the overall maximum rate of electron transfer from cyt. *c* into the electron acceptor Cu_A_.

The fitted *K*_d_ of 15 mM for the ATP binding to Cox13 observed in NMR titrations (Fig. 5) occurs in the same region as the tentative *K*_d_ fitted for ATP effects on turnover of Cyt*c*O (Additional file 1: Figure S7), and we also see clear effects on cyt. *c* titrations at 5 mM ATP (Fig. 4). The higher values obtained in NMR titrations could be due to the presence of added Arg and Glu in the NMR buffer (to stabilize the protein for the long NMR experiments), which presumably shields electrostatic interactions. We note that these ATP binding effects on Cox13 (and Cyt*c*O) occur in the mM region, which is consistent with the concentrations measured for cytosolic ATP in mammalian and yeast mitochondria (5 mM) and the ADP concentration is typically 5–10 times lower ([44, 45], with the lower range of ATP/ADP ratios observed in yeast). This means that even if ADP binds with similar *K*_d_ (Additional file 1: Figure S8), the much lower concentrations of ADP would favor ATP binding.

## Conclusion

In summary, our study provides structural information for an important subunit of Cyt*c*O that was used for identifying residues that are affected by the binding of ATP. Our data suggest that Cox13 houses an allosteric binding site for ATP in the intermembrane space and provides additional and significant insight into the Cyt*c*O enzymatic process and regulation.

## Methods

### Protein expression and purification

The gene of Cox13 from *S. cerevisiae* was cloned into pET-28a (Novagen) and expressed in *E. coli* Rosetta 2 as a His-tagged fusion (at C terminus) protein. The procedure used was similar to the one used to purify Rcf1 and Rcf2 [46, 47] with some minor differences in buffer composition. Cells were grown at 37 °C to an A_600_ of 0.5, induced with 1 mM IPTG, and allowed to express the protein for 12 h before harvesting. The media used was M9 minimal medium containing ^15^N ammonium chloride and/or ^13^C glucose (Cambridge Isotopes Laboratory). The bacterial pellet was re-suspended and passed through a French press, and the Cox13 inclusion bodies were purified under denaturing conditions (20 mM Tris, pH 8.0, 300 mM NaCl, 5 mM 2-mercaptoethanol, 5 mM imidazole, and 6 M guanidinium chloride) by affinity chromatography on Ni-NTA resin (Sigma). The purified denatured inclusion bodies were dialyzed against water to precipitate the inclusion bodies, which were then re-solubilized with 2 mL 8 M guanidinium chloride. The 2 mL re-solubilized inclusion bodies were slowly diluted into 20 mL refolding buffer (20 mM NaP_i_ pH 6.5, 300 mM NaCl, 500 mM arginine, 0.5 mM oxidized glutathione, 5 mM reduced glutathione, 10% glycerol, 1 mM EDTA, and 3 mM DPC) with stirring at 4 °C overnight. Next, the refolded Cox13 protein was concentrated and loaded onto a Superdex 200 10/300 column (GE Healthcare) pre-equilibrated with gel filtration buffer (20 mM NaP_i_ pH 6.5, 50 mM L-Arg, 50 mM L-Glu [48], 1 mM DTT, 3 mM DPC, or DPC-d_38_). The fractions containing Cox13 protein were concentrated, and the concentration was determined by a Lowry assay.

### Circular dichroism spectroscopy

For CD experiments, a Cox13 NMR sample was exchanged into a CD compatible buffer using a PD10 column (GE Healthcare), followed by concentration using a centrifugal concentrator with 10 kDa molecular weight cut-off. The CD buffer consisted of 30 mM DPC, 25 mM KP_i_ pH 6.5, 1 mM DTT, and 50 mM K_2_SO_4_ to replace free Arg and Glu in the NMR buffer. The final CD samples consisted of 7.6 or 135 μM Cox13, as determined from the A_280_, for measurements of a single CD spectrum and for the ATP titration series, respectively. Quartz cuvettes with path lengths of 1 mm and 50 μm where used in the former and latter experiments, respectively.

CD spectra were recorded on a Chirascan spectrometer (Applied Photophysics). The temperature was set to 298 K using an external water bath. Spectra were obtained in 1 nm steps from 260 to 190 nm with a measurement time of 0.5 s at each wavelength. Ten scans were acquired and averaged. For the ATP titration experiments, the final ATP concentrations were 0, 1, or 2.5 mM in CD buffer. The collection of high-quality spectra at even higher concentration ratios was prohibited by the strong absorption of ATP in the lower wavelength part of the spectrum. For each titration step, a background spectrum was recorded by adding the corresponding amount of ATP to the CD buffer only. Background spectra were subtracted from the ones with protein, and spectra were corrected for dilution upon ATP addition and normalized to units of mean residue molar ellipticity. Finally, CD spectra were smoothened through averaging within a three-point window function.

### NMR spectroscopy

All NMR data were obtained at 40 °C on Bruker 900 MHz or 800 MHz spectrometers equipped with cryogenic probes. Spectra were processed with the software TopSpin (Bruker) and analyzed using CcpNmr [49]. The concentration of ^15^N,^13^C-labeled Cox13 was 0.5 mM in 20 mM NaP_i_ pH 6.5, 50 mM L-Arg, 50 mM L-Glu, 1 mM DTT, and 30 mM DPC-d_38_ in 90% H_2_O/10% D_2_O. Backbone chemical shifts were assigned in a sequential manner from the following experiments: 2D ^1^H-^15^N TROSY-HSQC and 3D TROSY-versions of the HNCA, HN(CO)CA, HNCO, HN(CA)CO, HNCACB, and HN(CO)CACB spectra [50–52]. Sidechain proton and carbon chemical shifts were assigned using the following experiments: 3D (H)CCH-TOCSY, 3D CC(CO)NH, and 3D H(CCO)NH spectra [53, 54]. NOEs were obtained from 3D ^15^N-NOESY-HSQC (mixing time (τNOE) = 100 ms), 3D ^13^C-NOESY-HSQC (τNOE= 100 ms) and 3D SOFAST-^13^C-HMQC-NOESY-^13^C-HMQC (τNOE= 100 ms) spectra [55, 56]. The 3D F1-^13^C/^15^N-filtered, F3-^15^N-edited-NOESY-HSQC (τNOE= 200 ms) and 3D F1-^13^C/^15^N-filtered, F3-^13^C-edited-NOESY-HSQC (τNOE= 200 ms) spectra were acquired using a sample that was reconstituted with a 1:1 mixture of ^15^N-,^13^C-labeled Cox13 and unlabeled Cox13 to assign the intermolecular NOE contacts [25]. The mixing of the labeled and unlabeled sample was performed at the level of inclusion bodies.

^31^P experiments were performed on a Bruker 400 MHz spectrometer equipped with a BBO probe. The temperature was set to 298 K.

### Structure calculation

The ARIA program (version 2.3) [57] was used to help the assignment of short- and middle-range cross-peaks automatically. The program CNS (version 1.21) [26] was used to generate the structures through a simulated annealing protocol. The torsion angle restraints were determined from backbone chemical shift data using DANGLE [58]. The manually and automatically assigned unambiguous short-range and medium-range, as well as the manually assigned unambiguous long-range distance restraints were used for the monomer structure calculation. Monomer structures were calculated by 180 ps of torsion angle dynamics, followed by 72 ps of slow cooling in torsion angle space and 72 ps of slow cooling in cartesian space. In the next round of dimer structure calculation, two copies of the lowest-energy monomer structure were used to construct the dimer model by combining the unambiguous inter-monomer distance restraints derived from 3D F1-^13^C/^15^N-filtered, F3-^15^N-edited-NOESY-HSQC and 3D F1-^13^C/^15^N-filtered, F3-^13^C-edited-NOESY-HSQC spectra. A total of 100 structures were calculated and the 15 lowest-energy structures were selected to represent the structural ensemble. Protein structures were analyzed using PROCHECK [59] and displayed using Chimera [60]. Structural statistics are summarized in Additional file 1: Table S1.

### Paramagnetic spin-label titration

Water-soluble gadodiamide (Omniscan) was added from a 0.5 M stock solution into 500 μl ^15^N-labeled Cox13 with the final concentrations of 1 mM, 5 mM, and 10 mM. The detergent-soluble 16-DSA powder was dissolved in methanol, divided into aliquots corresponding to final concentrations of 1 mM, 5 mM, and 10 mM in the Cox13 sample, and methanol was air-dried away before adding to the protein. 2D ^1^H-^15^N-TROSY-HSQC spectra were recorded at 40 °C for each concentration of spin-label reagent, together with spectra before addition of spin-label to obtain reference resonance intensities. As a qualitative measure of a residue’s distance to a paramagnetic species, intensity ratios *I*/*I*_ref_ were calculated where *I* and *I*_ref_ are peak intensities in spectra with and without spin-label, respectively. Resonances that could not be assigned unambiguously by comparison to assigned reference spectra were excluded from the analysis, as well as resonances with large overlap, or large intensity increases after spin-label addition (i.e., due to decreasing exchange dynamics). Relative uncertainties in intensity ratios were calculated by assigning an arbitrary error to all peaks (5% of average reference spectrum peak intensity) followed by error propagation. To aid identification of trends in intensity ratios as a function of sequence, a weighted moving average was calculated, weighting by sequential distance (according to a Gaussian distribution with three residue halfwidth) and relative uncertainties in intensity ratios.

### Yeast strain construction and purification of Cyt*c*O

Two strain variants were used to purify Cyt*c*O, a W303 strain with a 6xHis-tag on Cox13 [61] and a BY4741 Cox13Δ strain with a Flag-tag on Cox6. The strain BY4741 *cox13*Δ Cox6Flag was constructed by first introducing a Flag-tag by exchanging the endogenous stop codon of the *COX6* open reading frame with a Flag-tag followed by a *HIS3* selection cassette. Subsequently, *COX13* was deleted by homologous recombination with a *KanMX4* selection cassette.

Yeast cells were grown on YP-media (2% peptone and 1% yeast extract, pH of the media adjusted to 5.5) supplemented with 2% glycerol.

Membranes from crushed yeast cells were used as starting material for the protein purification. Membranes were prepared by harvesting the yeast cells (5500×*g* for 5 min) followed by a wash in 50 mM KP_i_ buffer pH 7.4. The cell pellet was suspended in buffer (0.4 M sorbitol, 50 mM KP_i_ buffer pH 7.4, and 5 mM EDTA) at the ratio of 1:3 (w/v) and run twice through a cell disruptor (Constant Systems _LTD_) at 35000 psi. A few crystals of phenylmethanesulfonyl fluoride (PMSF) were added immediately after the crushing step. The cell homogenate was centrifuged at 5500×*g* for 10 min to remove unbroken cells. The supernatant was centrifuged at 138,000×*g* for 1 h to pellet membranes. The membranes were washed once in buffer containing 100 mM KCl and 50 mM KP_i_ buffer pH 8 (6xHis-tag variant on Cox13) or pH 7.4 (Cox13Δ variant) and then homogenized in wash buffer to a protein concentration ~20 mg/ml and flash-frozen in liquid nitrogen until further use.

His-tagged CytcO was purified according to a procedure by Meunier et al. [61] with the following modifications: during solubilization, the membranes were diluted to 2 mg protein/ml, dodecyl-β-D-maltoside (DDM) concentration was 1.5% (w/v) and the buffer contained an additional 100 mM KCl. Solubilization lasted 1 h at 5 °C with mild stirring. After solubilization, the solution was run through a Bio-Rex 70 cation exchanger (BIO-RAD) to remove cytochrome *c* from the membranes. The resin was converted to the potassium form. Five grams of cation exchanger/100 ml of solution was used. After cation exchange, the solubilized membranes were loaded at ~0.2 ml/min on a Ni sepharose 6 fast flow matrix (GE Healthcare) equilibrated with 20 mM KP_i_ buffer pH 8, 150 mM KCl and 0.035% DDM. The column was washed with 5 column volumes of the same buffer containing 10 mM imidazole. Elution was performed with 4 column volumes of buffer with 40 mM imidazole followed by 4 column volumes containing 100 mM imidazole. The DEAE sepharose column step was omitted, and the eluted fractions were concentrated and pooled. During concentration, buffer exchange was performed to reduce the imidazole concentration to the μM range.

Flag-tagged cytochrome *c* oxidase was purified with the following method: solubilization was performed in the same way as for the His-tagged CytcO with the exception that the pH of the buffer was 7.4. After solubilization, the solution was diluted in buffer (100 mM KCl, 50 mM KP_i_ buffer pH 7.4) to a DDM concentration of 0.2%. If the volume exceeded 100 ml, the solution was concentrated below that volume. The solution was loaded on 2 ml of anti-FLAG M2 affinity gel equilibrated with buffer (150 mM KCl, 20 mM KP_i_ buffer pH 7.4, and 0.035% DDM). The column was then washed with 20 column volumes of the equilibration buffer followed by elution with 5 column volumes of the same buffer with the addition of 0.1 mg/ml 1xFlag peptide. The eluted oxidase was concentrated with a 100 kDa cutoff filter (Merck Millipore) and frozen in liquid nitrogen.

### Cytochrome *c* oxidase activity measurements in the presence and absence of nucleotides

Cyt*c*O activity was measured by monitoring the oxygen consumption of purified yeast enzyme from wildtype and ΔCox13-variant as a function of added yeast cyt. *c* both in the absence and presence of 5 mM ATP. The activity was also measured in the presence and absence of ATP and ADP at varying concentrations. The measurements were performed with a Clark-type electrode (Hansatech Instruments). The buffer was composed of 25 mM KP_i_ pH 6.2 or 100 mM KP_i_ pH 7.0 and 0.035% DDM, and the measurement volume was 1 ml after the final addition. Ascorbate (10 mM), TMPD (0.1 mM), and cyt. *c* from yeast (50 μM, or for the titrations at the concentrations indicated) were used as electron donors. The nucleotide stock solutions were dissolved in equimolar amounts of KP_i_ buffer at pH 6.2 or pH 7.0. The background rate after addition of the reductants was subtracted from the final rates.

### ATP titration

The ^15^N-labeled Cox13 sample was 0.5 mM in a buffer of 20 mM NaP_i_, pH 6.5, 50 mM L-Arg, 50 mM L-Glu, 1 mM DTT, and 30 mM DPC in 90% H_2_O/10% D_2_O. 0.5 M stock solution of ATP (Sigma) in the same buffer as Cox13 was titrated into the Cox13 sample that provided the following molar ratios of ligand to protein: 2:1, 5:1, 10:1, 25:1, 50:1, and 100:1. A 2D ^1^H-^15^N-HSQC spectrum was collected at each titration point. The weighted total shift change, the chemical shift perturbation (CSP), for each peak was calculated by $\mathrm{CSP}=\sqrt{{\left(\Delta  \delta H\right)}^2+{\left(\alpha \Delta  \delta N\right)}^2}$, where ΔδH and ΔδN are the chemical shift differences for ^1^H and ^15^N, respectively, and *α* a shift weighting factor of 0.2 for Gly and 0.14 for other residues [39].

^31^P NMR was used to monitor the effect of Cox13 on ATP. The sample consisted of 600 μL 22 μM unlabeled Cox13 in NMR-buffer (25 mM KP_i_ pH 6.5, 50 mM Arg, 50 mM Glu, 1 mM DTT, 10% D_2_O) with 45 mM DPC. A 100-mM ATP stock solution in 100 mM NaP_i_ pH 6.5 was used for the titrations. 1D ^31^P spectra were recorded for [ATP]/[Cox13]-ratios of 1, 5, 10, 25, 50, 100, 250, and 500. For each ATP titration step to Cox13, a background spectrum was recorded on the corresponding ATP concentration in NMR buffer with 30 mM DPC but without protein. Due to the large range of ATP concentrations, experimental time varied between several hours down to a couple of minutes per spectrum to achieve approximately the same level of signal to noise in all spectra. The recycle delay was set to 1 s.

### HADDOCK docking procedure

ATP was docked to Cox13 using HADDOCK [62], and the interaction was guided based on the results of the NMR chemical shift perturbation experiments. Residues R81, H83, Y96, and I125, which displayed large CSPs were considered active in binding, and their amide protons were unambiguously constrained to a distance of 2.5 ± 2.5 Å from a central atom of the ATP molecule (C5'). The docking was performed simultaneously to all subunits A of the NMR ensemble and subsequently to all subunits B. To allow for a broad search of Cox13’s conformational space, the region between α2 and α3, and the C-terminal loop were considered fully flexible. 10,000 structures were initially generated, out of which 1000 and 500 were refined and water-refined, respectively. Apart from the aforementioned settings, default parameters were used throughout all docking procedures.

## Supplementary Information

**Additional file 1: Figure S1.** Purification and characterization of Cox13 in micelles. **Figure S2.** Representative sequential backbone assignment of Cox13. **Figure S3.** Representative slices in the 3D ^15^N, ^13^C-edited NOESY spectra. **Figure S4.** Representative slices from the 3D ^15^N-edited/filtered NOESY spectra. **Figure S5.** Paramagnetic spin-label titrations. **Figure S6.** Catalytic turnover of wildtype and Cox13Δ Cyt*c*O. **Figure S7.** Turnover in wildtype and Cox13Δ Cyt*c*O as a function of added yeast cytochrome *c*. **Figure S8.** Interaction of Cox13 with ATP. **Figure S9.** Interaction of ATP with Cox13. **Figure S10.** Interaction of Cox13 with ADP. **Table S1.** Summary of structural ensemble statistics. **Table S2.** Intermolecular NOEs.

**Additional file 2.** Excel file with data related to Fig. 4.

**Additional file 3.** Original picture related to Additional file 1: Figure S1.

**Additional file 4.** Excel file with data for Additional file 1: Figure S5.

**Additional file 5.** Excel file with data for Additional file 1: Figure S6.

**Additional file 6.** Excel file with data for Additional file 1: Figure S10.

## Data Availability

All data generated or analyzed during this study are included in this published article, its supplementary information files, and publicly available repositories. Accession codes: The structural restraints and structure coordinates have been deposited with accession codes 6ZDB (to PDB) and 34522 (to BMRB), respectively.

## Abbreviations

Cyt*c*O: Cytochrome *c* oxidase
cyt.: Cytochrome
DDM: *N*-dodecyl-β-d-maltoside
TMPD: N,N,N′,N′-tetramethyl-p-phenylenediamine
P_i_: Inorganic phosphate (HPO_4_^2-^ and H_2_PO_4_^-^)
CSP: Chemical shift perturbation
TM: Transmembrane
