# Supplementary material for: NMR structural analysis of the yeast cytochrome c oxidase subunit Cox13 and its interaction with ATP
Source: BMC Biol. 2021 May 10;19:98. doi: 10.1186/s12915-021-01036-x (PMC8111780; doi:10.1186/s12915-021-01036-x)
Supplement: Supplementary file 1 — Additional file 1: Figure S1. Purification and characterization of Cox13 in micelles. Figure S2. Representative sequential backbone assignment of Cox13. Figure S3. Representative slices in the 3D 15N, 13C-edited NOESY spectra. Figure S4. Representative slices from the 3D 15N-edited/filtered NOESY spectra. Figure S5. Paramagnetic spin-label titrations. Figure S6. Catalytic turnover of wildtype and Cox13Δ CytcO. Figure S7. Turnover in wildtype and Cox13Δ CytcO as a function of added yeast cytochrome c. Figure S8. Interaction of Cox13 with ATP. Figure S9. Interaction of ATP with Cox13. Figure S10. Interaction of Cox13 with ADP. Table S1. Summary of structural ensemble statistics. Table S2. Intermolecular NOEs. [file 12915_2021_1036_MOESM1_ESM.pdf]

Additional file 1: Supplementary figures and tables for manuscript:

"NMR structural analysis of the yeast cytochrome *c* oxidase subunit Cox13 and its interaction with ATP"

Shu Zhou[1b], Pontus Pettersson[b], Markus L. Björck[b], Hannah Dawitz, Peter Brzezinski, Lena Mäler*, Pia Ädelroth*

Affiliation: Department of Biochemistry and Biophysics, Stockholm University, Sweden

[1] Current address: High Magnetic Field Laboratory, Hefei Institutes of Physical Science, Chinese Academy of Sciences, China.
[b] Equal contribution

Corresponding Authors: pia.adelroth@dbb.su.se, lena.maler@dbb.su.se

This file contains:

Supplementary figures 1-10

Supplementary table 1-2

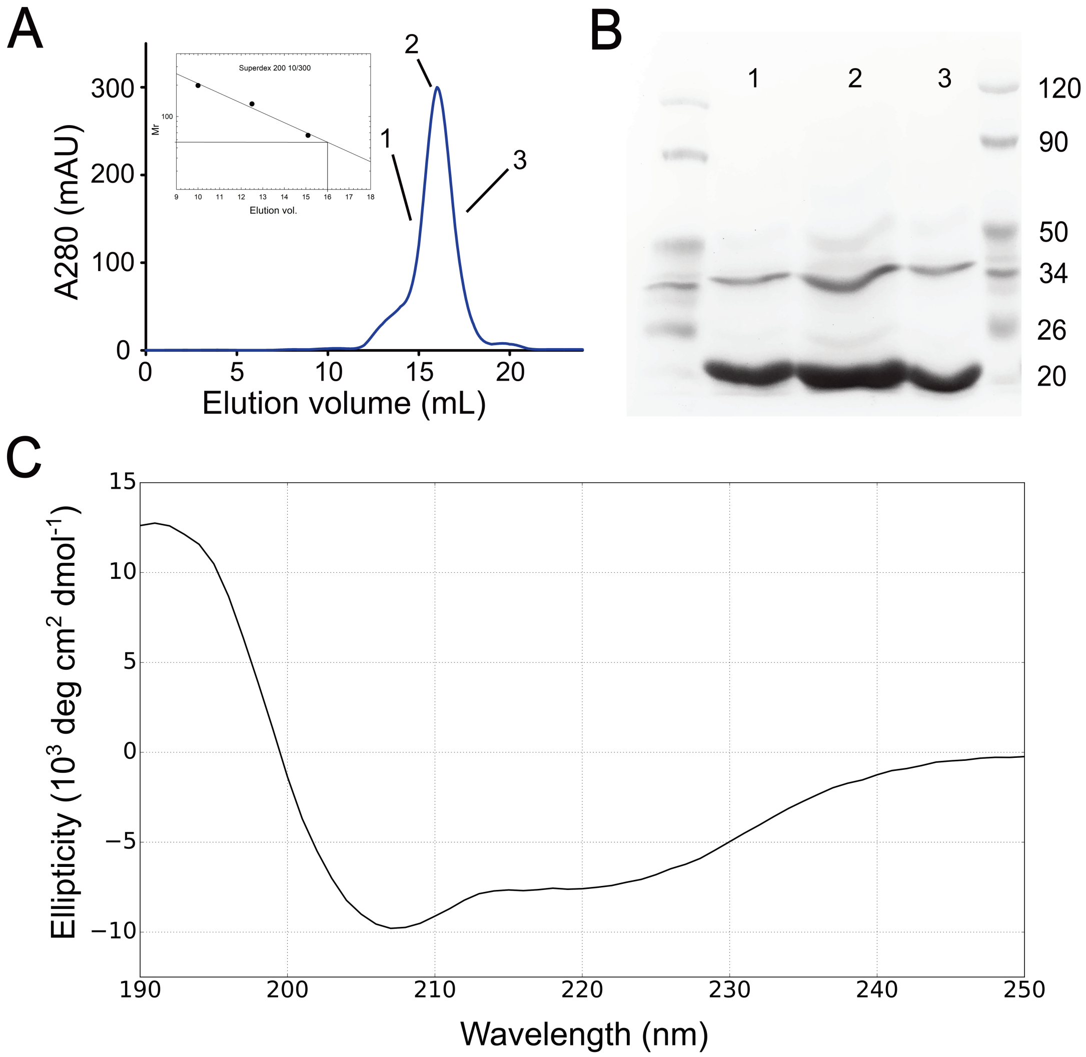

Figure S1 – Purification and characterization of Cox13 in micelles.

(A) Elution peak of Cox13 from a Superdex 200 10/300 GL column in 20 mM NaP_i pH 6.5 containing 50 mM L-Arg, 50 mM L-Glu, 1 mM DTT and 3 mM DPC. The inset shows a calibration with BSA and indicates that the apparent MW of the Cox13 micelle is 55-60 kDa. (B) SDS–PAGE analysis of the Cox13 elution peak showing a Cox13 monomer band at ~16 kDa and also a dimer band at ~32 kDa. The lane numbers correspond to the elution fraction numbers indicated in (A) For original picture, see Additional file 3. (C) CD spectrum of Cox13, assayed in 30 mM DPC, 25 mM KP_i pH 6.5, 1 mM DTT, and 50 mM K_2SO_4.

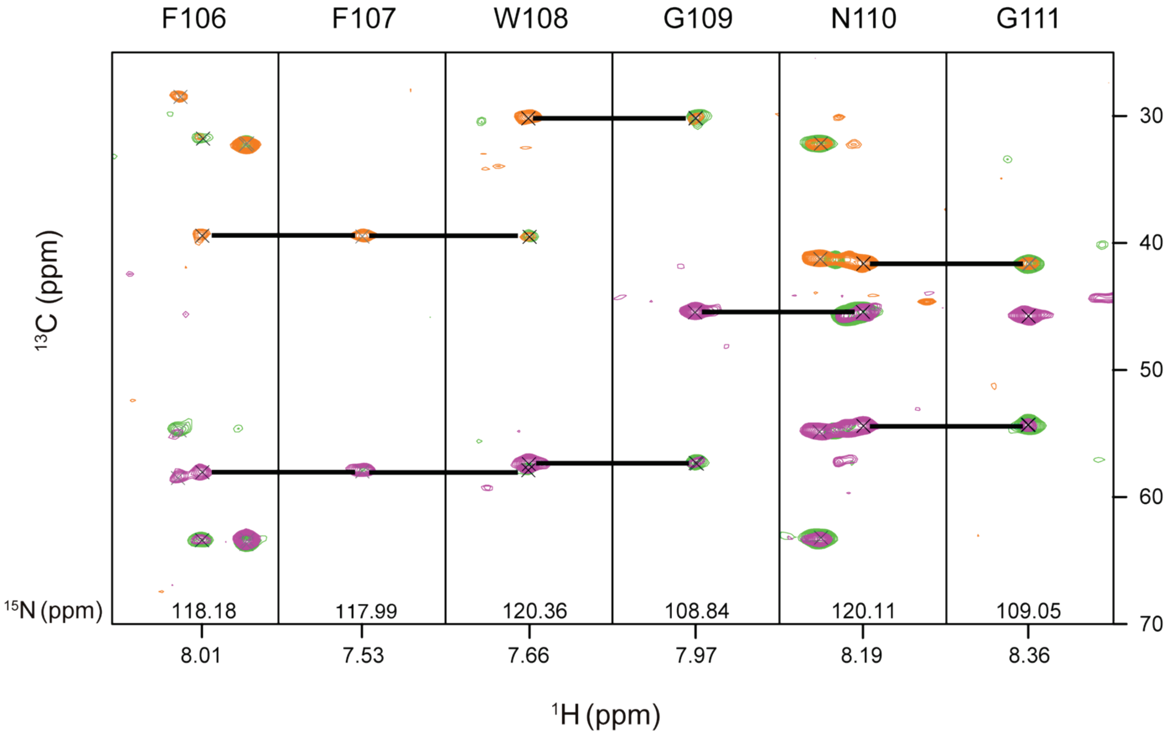

Figure S2 – Representative sequential backbone assignment of Cox13.

Strips for residues F106-G111 from the overlaid 3D-HNCACB (C^{α} in orange and C^{β} in pink) and 3D-HNCOCACB (resonance in green) spectra of [^{15}N, ^{13}C]-labelled Cox13 at a concentration of 0.4 mM in 20 mM NaP_i pH 6.5, containing 50 mM L-Arg, 50 mM L-Glu, 1 mM DTT and 30 mM DPC, recorded at 40 °C and 900 MHz. The lines indicate sequential connectivities.

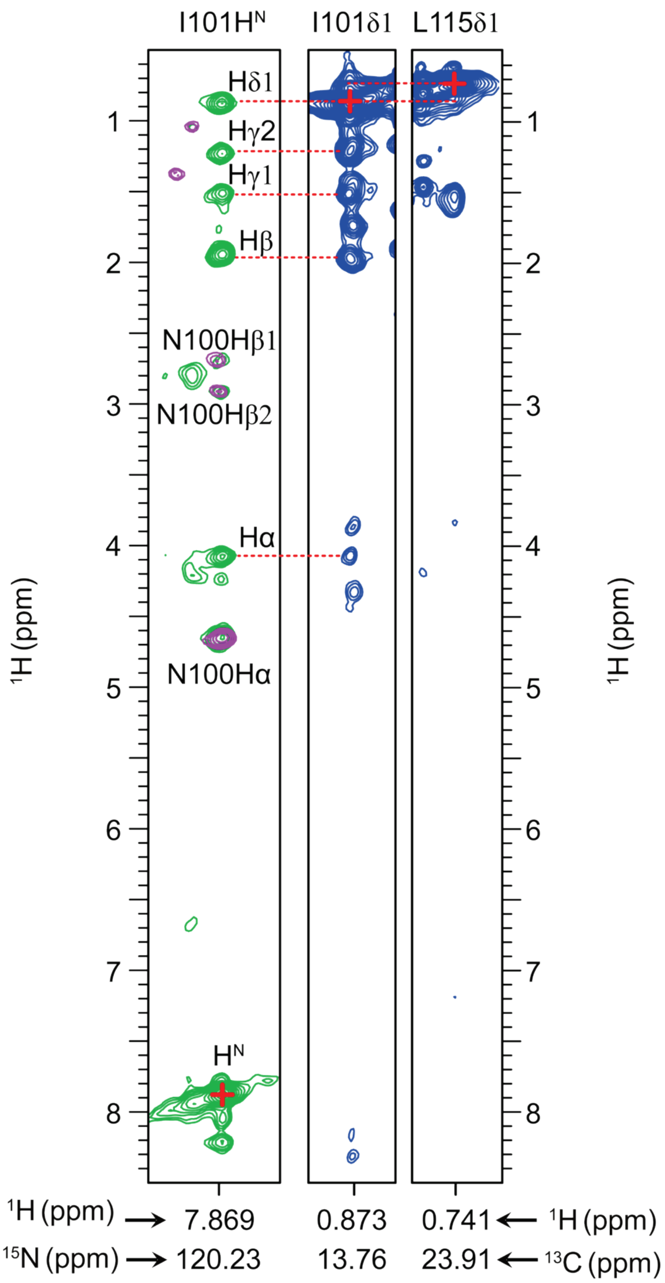

Figure S3 – Representative slices in the 3D ^{15}N, ^{13}C-edited NOESY spectra.

Example of an overlaid ^{1}H-^{1}H slice taken from the 3D ^{15}N-edited NOESY spectrum (green) and the 3D H(CCO)NH spectrum (pink). The assigned side-chain resonances of residue N100 and NOE resonances of residue I101 are indicated. The observed long-distance NOE correlation between residue I101 and L115 is shown in the 3D ^{13}C-edited NOESY spectrum (blue). The diagonal peak is marked with a red cross, and correlations are indicated by red dashed lines.

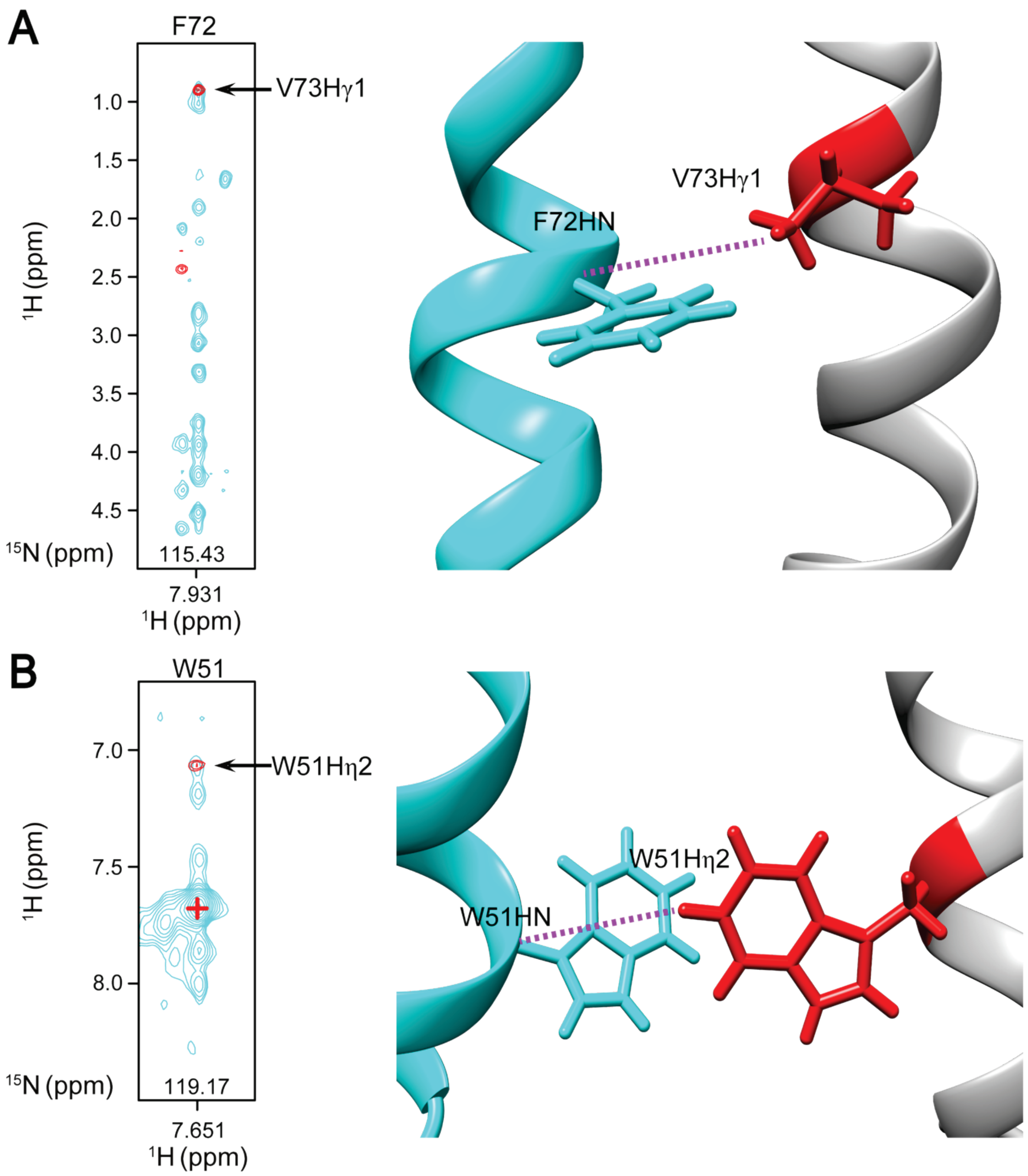

Figure S4 – Representative slices from the 3D ^{15}N-edited/filtered NOESY spectra.
Examples of overlaid ^{1}H-^{1}H slices for residue F72 (A) W51 (B) from the 3D ^{15}N-edited NOESY spectrum (cyan) and the 3D F1-^{13}C/^{15}N-filtered, F3-^{15}N-edited-NOESY spectrum (red). The corresponding inter-monomer NOE correlations are mapped onto the Cox13 structure by pink dashed lines. The monomer subunits of Cox13 structure are shown in cyan and grey, respectively. The diagonal peak in (B) is marked with a red cross.

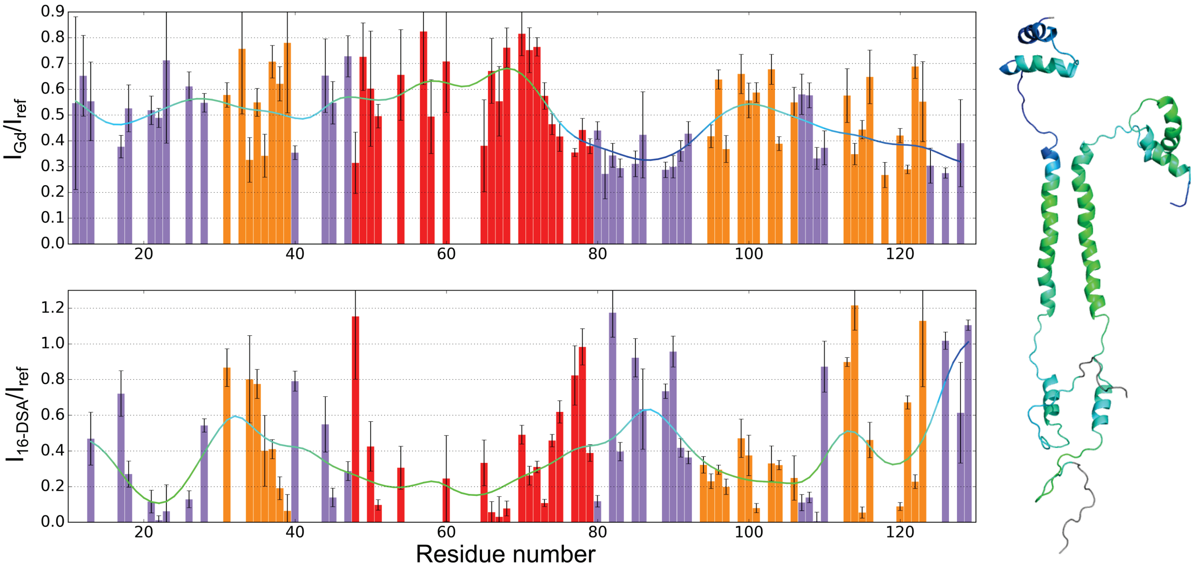

Figure S5 – Paramagnetic spin-label titrations.

Resonance intensity ratios as a function of sequence calculated from ^{1}H-^{15}N TROSY-HSQC spectra recorded on samples of 0.5 mM ^{15}N-labelled Cox13 in DPC micelles in the presence or absence of 5 mM gadodiamide (upper) or 5 mM 16-DSA (lower). For more details, see Additional file 4. Residues of regions assigned as random coil, soluble helix, and transmembrane helix are coloured purple, orange, and red, respectively. Error bars indicate relative uncertainties calculated by error propagation and curves are weighted moving averages, which take sequential distance and uncertainties into account. The weighted moving averages' colour scales go from blue to green to indicate regional partitioning into aqueous or hydrophobic environments, respectively. To illustrate the overall partitioning suggested by the Gd and DSA data, which is similar with only minor local differences, the Cox13 dimer structure has been colour-coded according to the values of the moving averages: the left subunit according to the upper (Gd) panel, and the right subunit according to the lower (DSA) panel.

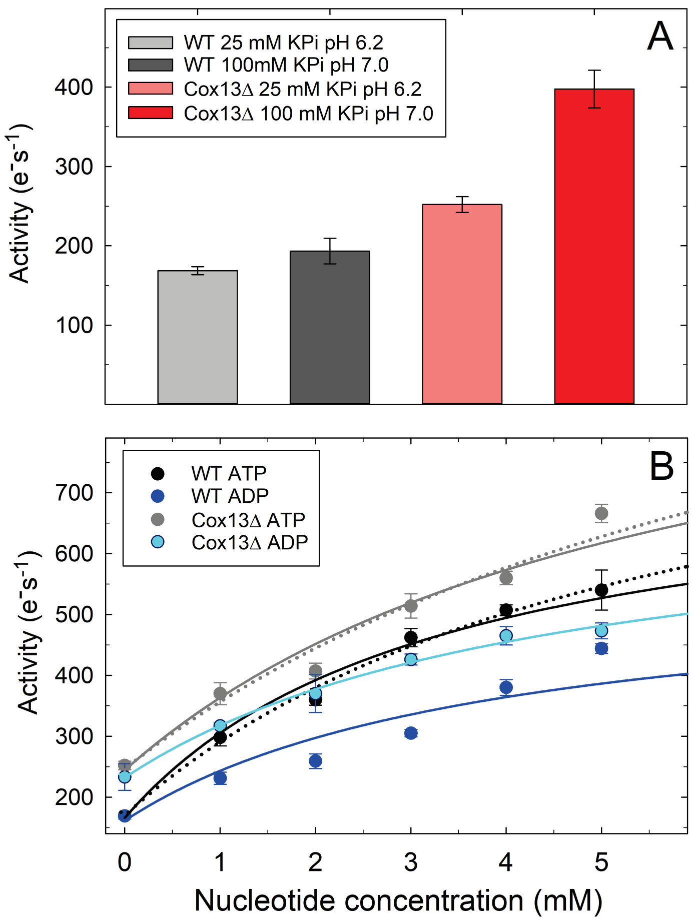

C

Sample	V_0 (e^-s^{-1})	K_d^1 ATP (mM)	$V_{max}^{1, ATP}$ (e^-s^{-1})	K_d^2 ATP (mM)	$V_{max}^{2, ATP}$ (e^-s^{-1})	K_d ADP (mM)	V_{max}^{ADP} (e^-s^{-1})
WT	**165**	**3.3**	**770**	5.6	970	***3.9**	**560**
ΔCox-13	**240**	**5.7**	**1040**	8.0	1250	**4.6**	**720**

*tentative fit only, data too scattered to give reliable parameters

Figure S6 – Catalytic turnover of wildtype and Cox13Δ Cyt*c*O.

(A) O_2-reduction activity in different KP_i buffers as indicated in the legend. (B) Cyt*c*O activity in the presence and absence of adenine nucleotides (ATP and ADP) at varying concentrations for the two different variants (wildtype Cyt*c*O with a histidine tag on Cox13 and ΔCox13 with a flag-tag on Cox6). Experimental conditions: T=25°C, buffer was 25 mM KP_i at pH 6.2 and 0.035% DDM. Electron donors were ascorbate (10 mM), TMPD (0.1 mM) and yeast cyt. *c* (50 µM). The ATP stock used was dissolved in equimolar amounts of the same KP_i buffer. Data points shown are averages and the error bars are standard deviations (*n*=5). For original data, see Additional file 5. The lines shown are fits to hyperbolic functions with the parameters indicated in the table. (C) The parameters used for the fits shown in B) where K_d^1 and K_d^2 for ATP refers to the two different fits (solid and dashed lines in B respectively) illustrating the range of possible fits. For the wildtype with ADP, the data was too scattered to give a reliable fit.

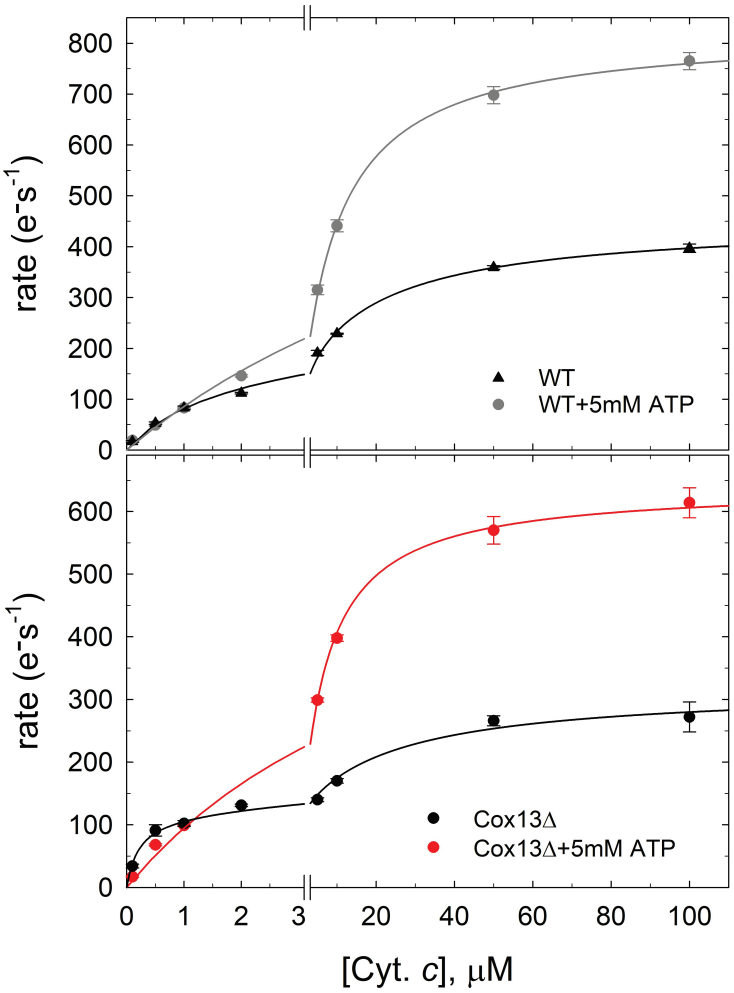

Figure S7 – Turnover in wildtype and Cox13Δ Cyt*c*O as a function of added yeast cytochrome *c*. The same data as in Figure 4 (main text) but plotted comparing the data with and without ATP in each variant directly and without normalising to the maximum activity at 100 μM cyt. *c*. The fits are the same as those shown in Figure 4.

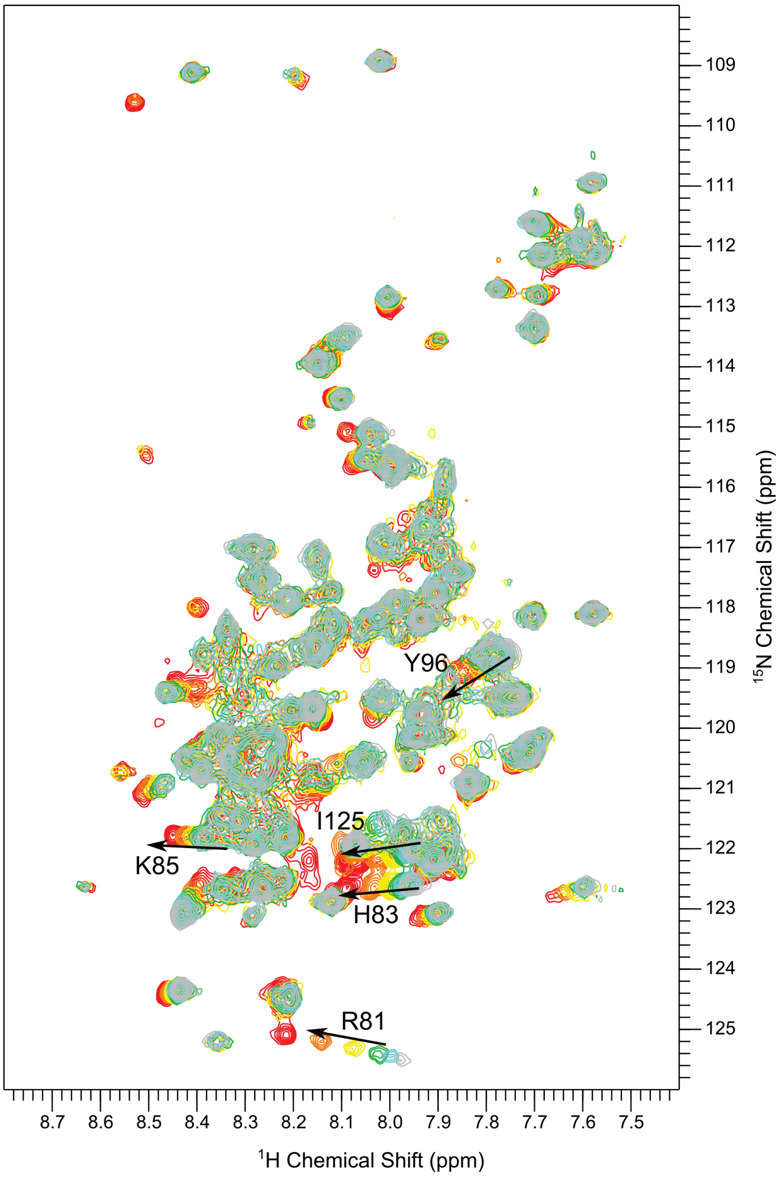

Figure S8 – Interaction of Cox13 with ATP.

Overlaid ^{15}N-HSQCs from an ATP titration to Cox13. Spectra are colour-coded with ATP/Cox13 ratios according to: 0 (grey), 5 (cyan), 10 (green), 25 (yellow), 50 (orange), and 100 (red). Arrows indicate directions of the chemical shift change for selected residues.

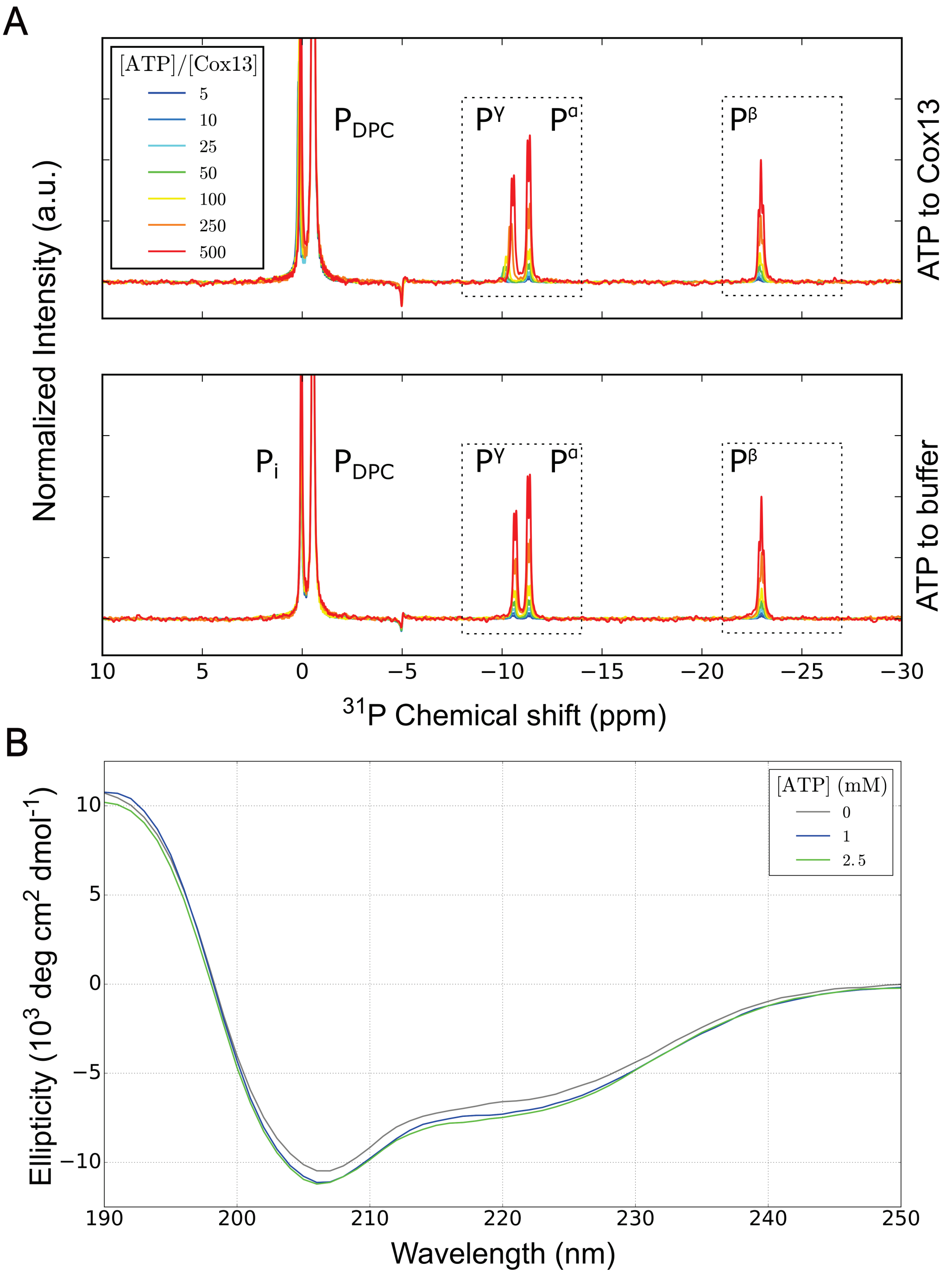

Figure S9 – Interaction of ATP with Cox13.

(A) Full width ^{31}P spectra from the ATP titration to Cox13 (upper panel) and to buffer containing detergent (lower panel). The chemical shift regions with the ATP resonances of Figure 5C are indicated with dashed rectangles. Spectra have been normalized according to the P_{DPC} peak height, and the y-axis scaled to emphasize the ATP resonances. (B) CD spectra of Cox13 at the ATP concentrations given in the legend.

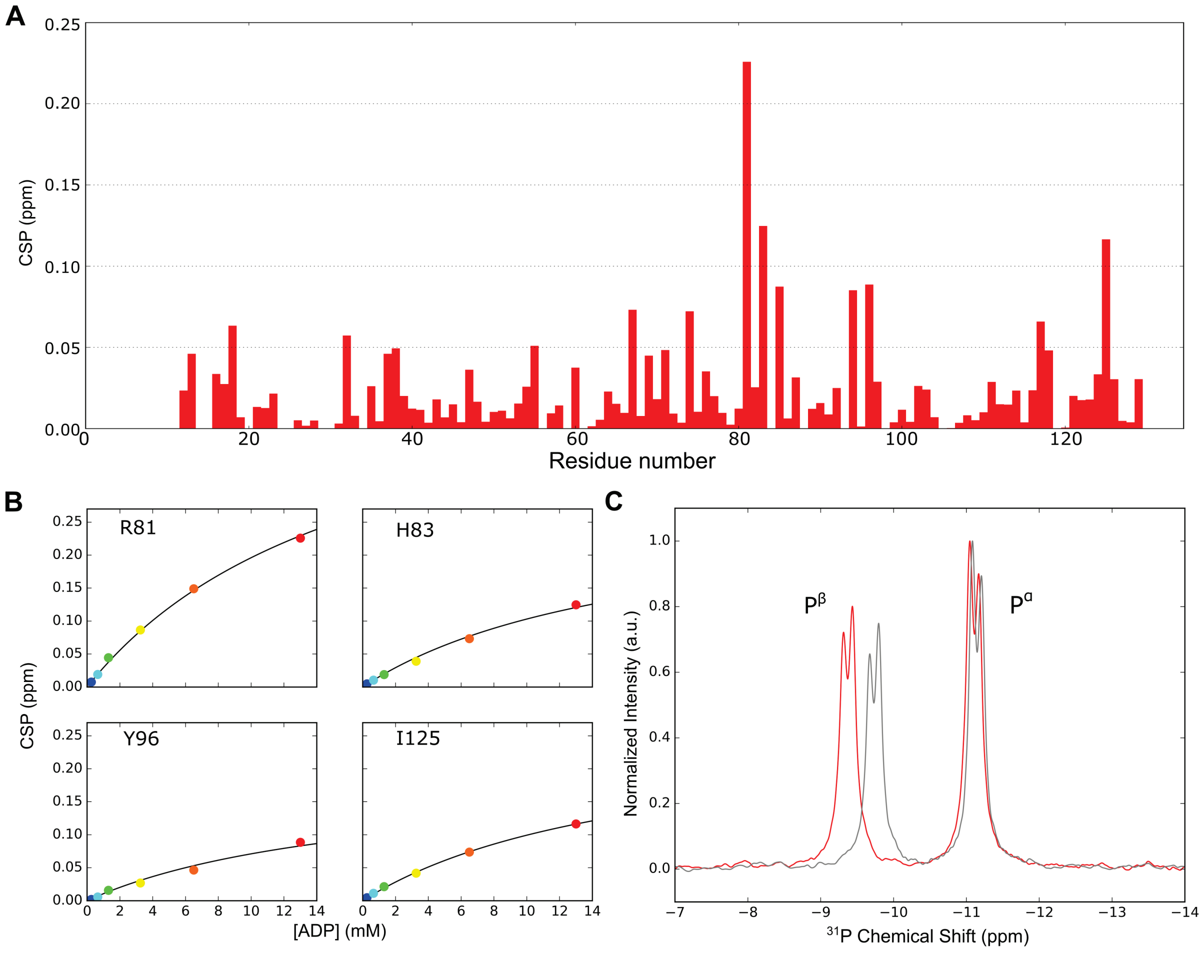

Figure S10 – Interaction of Cox13 with ADP.

(A) CSPs of Cox13 upon ADP addition. The CSPs as a function of primary sequence were calculated from peak positions in ^{15}N-HSQCs at the initial and final titration steps of [ADP]/[Cox13]=0 and 100 respectively. For more details, see Additional file 6. (B) CSP as a function of ADP concentration for selected residues. The curves are fitted as in Figure 5 (main text) with a global K_d = 17±2 mM. The fitted maximum CSPs for residues R81, H83, Y96 and I125 are 0.53±0.03, 0.28±0.02, 0.19±0.02, and 0.27±0.02 ppm, respectively. (C) Parts of ^{31}P spectra showing signals from the two phosphate groups of ADP in solutions with (red) and without (grey) Cox13. The red spectrum was recorded on the same sample as used in A. The grey spectrum was obtained on a sample where the ADP stock solution was added in the corresponding amount to NMR-buffer only.

**NMR distance and dihedral constraints** [(a)]	
Distance constraints	
Total NOE	1376
Intraresidual ($\|i-j\| = 0$)	758
Sequential ($\|i-j\| = 1$)	182
Medium-range ($1 < \|i-j\| \leq 4$)	306
Long-range ($\|i-j\| \geq 5$)	78
Intermolecular	52
Total dihedral angle restraints	
Phi	110
Psi	110
**Structure statistics** [(b)]	
Mean r.m.s.d from exp. restraints	
Distance constraints (Å)	0.026 ± 0.004
Dihedral angle constraints (°)	0.15 ± 0.02
Deviations from idealized geometry	
Bond lengths (Å)	0.002 ± 0.0002
Bond angles (°)	0.278 ± 0.01
Impropers (°)	0.10 ± 0.02
Average pairwise r.m.s. deviation (Å) [(c)]	
Heavy atoms	1.086
Backbone atoms	0.425
**Ramachandran plot**	
Most favored region	96.2%
Additionally allowed region	3.8%

Table S1 – Summary of structural ensemble statistics.

(a) The numbers of constraints refer to the dimer structure.

(b) Statistics are calculated and averaged over an ensemble of the 15 lowest energy structures out of 100 calculated structures.

(c) Statistics for TM region (residue 48-79)

Intermolecular	Subunit A		Subunit B	
**NOE nr.**	**Residue**	**Atom(s)**	**Residue**	**Atom(s)**
1	M50	$H\epsilon$	W51	$H\eta 2$
2	W51	HN	W51	$H\eta 2$
3	W51	$H\eta 2$	I54	$H\gamma 2$
4	W51	$H\eta 2$	I54	$H\delta 1$
5	I54	$H\gamma 2$	I54	$H\gamma 2$
6	V58	$H\beta$	V58	$H\gamma 1$
7	V58	$H\beta$	V58	$H\gamma 2$
8	V58	$H\gamma 1$	V58	$H\gamma 1$
9	V58	$H\gamma 1$	V58	$H\gamma 2$
10	V58	$H\gamma 1$	A62	$H\beta$
11	V58	$H\gamma 2$	V58	$H\gamma 2$
12	A62	$H\beta$	A62	$H\beta$
13	L65	$H\delta 1$	T66	$H\beta$
14	L65	$H\delta 1$	T66	$H\gamma 2$
15	L65	$H\delta 1$	N69	HN
16	L65	$H\delta 1$	N69	$H\beta$
17	L65	$H\delta 2$	T66	$H\beta$
18	L65	$H\delta 2$	T66	$H\gamma 2$
19	V68	$H\gamma 1$	N69	$H\beta$
20	V68	$H\gamma 1$	N69	$H\delta 2$
21	V68	$H\gamma 2$	N69	$H\delta 2$
22	F72	HN	V73	$H\gamma 1$
23	F72	$H\alpha$	V73	$H\gamma 1$
24	F72	$H\beta$	V73	$H\gamma 1$
25	F72	$H\epsilon$	V73	$H\gamma 1$
26	E76	HN	E76	$H\beta$

Table S2 – Intermolecular NOEs.

Assigned distance constraints between the Cox13 dimer subunits. The table lists one half of the symmetrical NOEs (the second half is obtained by swapping subunit indices A and B).
