# Supplementary figures and images for: NMR structural analysis of the yeast cytochrome c oxidase subunit Cox13 and its interaction with ATP
Source: BMC Biol. 2021 May 10;19:98. doi: 10.1186/s12915-021-01036-x (PMC8111780; doi:10.1186/s12915-021-01036-x)

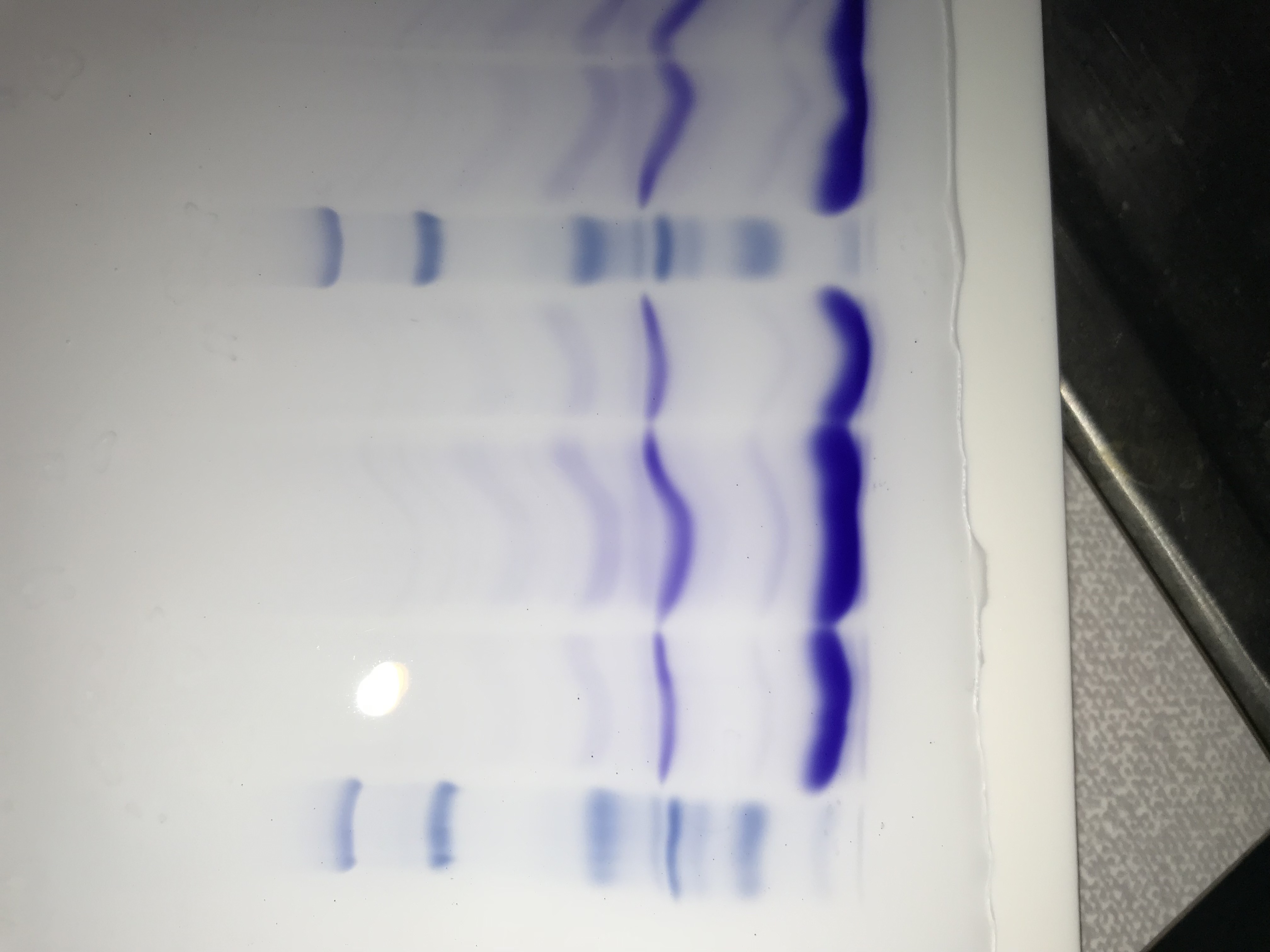

Supplement: Supplementary file 3 — Additional file 3. Original picture related to Additional file 1: Figure S1. [file 12915_2021_1036_MOESM3_ESM.jpg]
